# Coverage Gaps and Contraceptive Use Among Medicare Enrollees With Disabilities

**DOI:** 10.1001/jamanetworkopen.2025.17718

**Published:** 2025-06-25

**Authors:** Meghan Bellerose, Jacqueline Ellison, Maria W. Steenland, David J. Meyers, Monika Mitra, Theresa I. Shireman

**Affiliations:** 1 Department of Health Services, Policy, and Practice, Brown University School of Public Health, Providence, Rhode Island; 2 Department of Health Policy and Management, University of Pittsburgh School of Public Health, Pittsburgh, Pennsylvania; 3 Brown University Population Studies and Training Center, Providence, Rhode Island; 4 Lurie Institute for Disability Policy, Heller School for Social Policy and Management, Brandeis University, Waltham, Massachusetts

## Abstract

**Importance:**

Medicare is the primary health insurance payer for 1.5 million reproductive-aged women with disabilities, yet it is the only major form of US health insurance that is not required to cover contraceptives for pregnancy prevention.

**Objective:**

To evaluate whether Medicare’s contraceptive coverage gaps were associated with reduced use of contraceptives by enrollees with disabilities.

**Design, Setting, and Participants:**

In this national, cross-sectional study, traditional Medicare (TM), Medicare Advantage (MA), and Medicaid claims from female enrollees aged 20 to 49 years receiving Social Security Disability Insurance or Supplemental Security Income from January 1, 2016, to December 31, 2020, were linked. The propensity score–weighted probability of contraceptive use by public insurance type was estimated, then the association between gaining contraceptive coverage through a transition from Medicare to dual Medicare-Medicaid enrollment and contraceptive use was evaluated using a staggered-entry difference-in-differences design. Data were analyzed from December 3, 2024, to April 5, 2025.

**Exposures:**

Public insurance enrollment in TM, MA, dual TM-Medicaid, dual MA-Medicaid, or Medicaid.

**Main Outcomes and Measures:**

Monthly use of permanent contraceptives, long-acting reversible contraceptives (intrauterine device and implant), and short-acting contraceptives (injectable and oral contraceptives, patch, and ring).

**Results:**

A total of 51 501 303 monthly observations from 1 606 129 women were included in the analysis. Mean (SD) age was 35.93 (8.58) years; 1.8% of monthly observations were from Asian women, 30.7% from Black women, 13.0% from Hispanic women, 52.6% from White women, and 1.9% from multiracial women or women identifying as another race and ethnicity not reported on previously. Those enrolled in TM and MA were more often older and non-Hispanic White compared with those dual enrolled or enrolled in Medicaid. The estimated monthly probability of use of any contraceptive method was lowest among TM (4.9%; 95% CI, 4.9%-4.9%) and MA (6.6%; 95% CI, 6.5%-6.6%) enrollees, followed by Medicaid (11.0%; 95% CI, 11.0%-11.0%), dual MA-Medicaid (11.3%; 95% CI, 11.3%-11.4%), and dual TM-Medicaid (13.1%; 95% CI, 13.0%-13.1%) enrollees. Gaining contraceptive coverage through dual enrollment was associated with an increase of 3.9 (95% CI, 3.5-4.3) percentage points (35%) in use of any contraceptive method, with the largest increase in use of short-acting methods at 2.6 (95% CI, 2.3-3.0) percentage points (45%).

**Conclusions and Relevance:**

In this cross-sectional study of contraceptive use in the Medicare program, gaining contraceptive coverage through dual Medicare-Medicaid enrollment was associated with increased contraceptive use among disabled Medicare enrollees, suggesting that Medicare’s coverage gaps pose a financial barrier to desired contraceptive use. Given these findings, Medicare should be required to cover all US Food and Drug Administration–approved contraceptive methods without cost-sharing. Doing so would align Medicare’s coverage requirements with those of Medicaid, private insurance plans, and TRICARE.

## Key Points

**Question:**

Is Medicare's limited coverage of contraceptive methods associated with reduced contraceptive use among enrollees with disabilities?

**Findings:**

In this cross-sectional study of 1.6 million reproductive-aged women with disabilities, those enrolled in Medicare, who are subject to out-of-pocket costs for contraceptives, had lower contraceptive use compared with those enrolled in Medicaid or dual enrolled, who are not subject to those costs. Gaining contraceptive coverage through a transition from Medicare to dual enrollment was associated with increased contraceptive use.

**Meaning:**

These findings suggest that Medicare’s exemption from federal contraceptive coverage requirements and resulting coverage gaps are a barrier to desired contraceptive use among enrollees with disabilities.

## Teaser

This cross-sectional study assesses whether Medicare's contraceptive coverage policies are associated with limited contraceptive use among enrollees with disabilities.

## Introduction

The ability to control when and whether to become pregnant is central to reproductive autonomy and health, yet women with disabilities face substantial barriers to contraceptive access. In the US, women with disabilities are 32% less likely to use any contraceptive method compared with women without disabilities.^1,2^ The cost of contraceptives is a commonly reported barrier to use among disabled women.^3,4^ In 2024, women with disabilities were more than twice as likely as women without disabilities to report that they stopped using a contraceptive method because they could not afford it.^4^

Several pieces of federal legislation have expanded access to free contraception.^5^ In 1972, state Medicaid programs were required by federal law to cover all US Food and Drug Administration–approved contraceptives without cost-sharing or out-of-pocket costs to users via copayments and coinsurance.^6^ In 2012, the Affordable Care Act required that private insurance plans also cover all forms of contraception without cost-sharing.^7,8,9,10,11^ Most recently, in June 2023, TRICARE, the federal health insurance program for active duty military, veterans, and family members, ended cost-sharing for contraceptives.^12^ Today, Medicare is an outlier among US health insurance programs in its limited coverage of contraceptives.^13^

In January 2025, Medicare was the primary payer for roughly 1.5 million reproductive-aged women with disabilities, or approximately 13.2% of the 11.4 million disabled women aged 15 to 49 years in the US. Medicare enrollees can choose between traditional Medicare (TM) or a Medicare Advantage (MA) plan offering supplemental benefits. Neither TM nor MA plans are required to cover contraceptives for pregnancy prevention. Those with Part D or MA prescription drug benefits are typically eligible for coverage of short-acting methods (eg, oral contraceptive, patch, ring, injectable) with cost-sharing.^13^ In January 2024, TM and MA implemented coverage of long-acting reversible methods (eg, intrauterine devices [IUDs], implants) with cost-sharing. Previous research found that in 2019, before this change, women enrolled in TM had lower probabilities of incident contraceptive use compared with those enrolled in MA, likely as a consequence of greater MA coverage of long-acting methods.^14^ Neither TM nor MA cover permanent contraception (eg, tubal ligation, vasectomy) for pregnancy prevention, even when a clinician determines that a future pregnancy would endanger the enrollee’s health.^13^ For the roughly 60% of TM and MA enrollees who are simultaneously enrolled in Medicaid (“dual enrolled”) due to low incomes, Medicaid covers contraceptives without cost-sharing.^14^

The aim of this study was to assess the degree to which Medicare’s contraceptive coverage policies restrict enrollees’ contraceptive use. We described differences in contraceptive use between women with disabilities enrolled in TM, MA, or Medicaid or dual enrolled, then evaluated the association of gaining contraceptive coverage through a transition from Medicare to dual Medicare-Medicaid enrollment with contraceptive use.

## Methods

### Language and Approvals

In this cross-sectional study, we use both person-first (eg, *people with disabilities*) and identify-first (eg, *disabled people*) language to honor the different preferences of disability communities.^15^ This study was deemed exempt from full review and informed consent by Brown University’s Institutional Review Board as a research project subject to approval by a federal department or agency and designed to study, evaluate, or improve a public benefit or service program. We followed the Strengthening the Reporting of Observational Studies in Epidemiology (STROBE) reporting guidelines.

### Data and Cohort

Sample construction is detailed in eMethods 1 in Supplement 1. We used TM, MA, and Medicaid claims data from all 50 US states and Washington, DC, from January 1, 2016, to December 31, 2020, including beneficiary summary files and inpatient, outpatient, physician, and pharmaceutical claims. TM and MA claims were drawn from a 20% random national sample of enrollees and Medicaid claims from a 100% national sample. These files include consistent person identifiers, allowing us to link them and capture transitions between insurance types.

Our sample included female enrollees aged 20 to 49 years receiving Social Security Disability Insurance (SSDI) or Supplemental Security Income (SSI) who were enrolled in TM, MA, or Medicaid for at least 1 month between January 2016 and December 2020. Medicare is available to people younger than 65 years after 2 years of receiving SSDI for a disability that limits their ability to work and is expected to last for at least 1 year. To match this population, we restricted the Medicaid sample to women receiving SSDI or SSI, which use the same set of disability conditions for eligibility. We considered women ineligible for contraceptives following a hysterectomy and during pregnancy or within 2 months post partum. We removed individuals missing variables used in the models described below. A flow diagram of study inclusion is found in eFigure 1 in Supplement 1.

### Exposures

We examined 5 forms of public health insurance: TM alone, MA alone, dual TM-Medicaid, dual MA-Medicaid, and Medicaid alone. We excluded women with partial dual enrollment (receiving Medicaid coverage of Part A or B premiums, but no other Medicaid benefits).^16^

### Outcomes

We examined monthly receipt of the following contraceptive methods observable in claims: female permanent contraceptives (tubal ligation and salpingectomy), long-acting reversible contraceptives (IUD and implant), and short-acting contraceptives (injectable and oral contraceptives, patch, and ring). We excluded methods available over the counter during the study period, including condoms. We used codes defined by the Office of Population Affairs, including *International Statistical Classification of Diseases and Related Health Problems, Tenth Revision* (*ICD-10*), diagnosis related group, and *Current Procedural Terminology* codes to identify provision or removal of methods during inpatient or outpatient health visits (eMethods 2 and eTable 1 in Supplement 1) and National Drug Codes to identify prescription methods from pharmaceutical claims (eMethods 2 and eTable 2 in Supplement 1).

We recorded female enrollees with claims for permanent contraceptives as using those methods for all future months and those with claims for implants and IUDs (without subsequent removal) as using those methods for 3 and 5 years, respectively, based on efficacy lengths of the most common methods.^17,18^ We used days’ supply from pharmaceutical claims to identify length of injectable, pill, patch, and ring prescriptions.

### Statistical Analysis

#### Contraceptive Use by Insurance Type

Data were analyzed from December 3, 2024, to April 5, 2025. We estimated the monthly probability of contraceptive use among female enrollees by insurance type. To account for differences in the characteristics of those eligible and ineligible for Medicaid, we used inverse probability of treatment weighting.^19,20^ We created propensity scores using logistic regression models with the binary outcome of Medicaid enrollment and the following factors: age category, race and ethnicity (Asian, Black, Hispanic, White, multiracial, and other), county-level MA plan penetration, and 3 zip code–level measures from the US Census Bureau, specifically median household income, poverty rate, and 4-year college degree rate. We included race and ethnicity as proxies for institutional and interpersonal racism, which affects one’s likelihood of being eligible for Medicaid, enrolling in Medicaid, and accessing contraceptive counseling and care. We evaluated the performance of propensity scores in achieving covariate balance, visually assessed the common support assumption, and achieved strong covariate balance based on standardized mean differences below 0.1 (eMethods 3, eTables 3-5, and eFigures 2 and 3 in Supplement 1). We included propensity scores in logistic regression models with the outcome of monthly contraceptive use and the exposure of insurance type and presented results as marginal effects. We evaluated differences between groups using χ^2^ tests, with a threshold of 2-sided *P* = .05 for statistical significance.

#### Association of Gaining Coverage With Contraceptive Use

Next, we evaluated the association of gaining contraceptive coverage through a transition from Medicare to dual enrollment with contraceptive use. We restricted our sample to a treatment group who transitioned from Medicare (TM or MA) to dual enrollment (TM-Medicaid or MA-Medicaid) and a control group continuously enrolled in Medicare alone. Common situations when Medicare enrollees become newly dual enrolled include loss of household income, changes in their states’ Medicaid eligibility levels (eg, through the Affordable Care Act Medicaid expansions), moving to states with lower eligibility thresholds, pregnancy, or changes in household composition that reduce household income as a percentage of the federal poverty level (eg, marriage, adoption).

We used a staggered-entry difference-in-differences (sDID) design to account for variation in treatment timing, or the month during which transitions occurred (eMethods 4 in Supplement 1). We used estimators from Callaway and Sant’Anna^21^ to eliminate biased comparisons of transitions later in the study period to earlier transitions. We assessed contraceptive use for the 12 months before and after each transition. For the 9.8% of women with multiple transitions during the study period, we randomly selected a single transition to evaluate.

To account for potential variation in contraceptive use from having more months of continuous insurance enrollment, we assigned each control group member a proxy transition month and corresponding before and after periods to match that of a treatment group member. Following prior work,^22^ we did so by calculating the time difference between each treatment group member’s Medicare enrollment month and their transition month, randomly assigning those time differences to control group members based on the frequency distribution and adding the time differences to each control group member’s Medicare enrollment month.

We recalculated propensity scores within our subsample with the binary outcome of being in the treatment vs control group and applied them in sDID models. We included individual fixed effects so that estimates reflected changes in contraceptive use within the same individuals before and after transitions and clustered standard errors at the individual level to account for correlation of repeated measures.^23^ We included monthly fixed effects to account for differences in Medicaid eligibility thresholds across time.^24^ Our treatment effect represented the mean difference in contraceptive use before vs after a Medicare to dual enrollment transition relative to remaining enrolled in Medicare alone. We tested model assumptions and provided evidence of parallel contraceptive use patterns in the pretransition period (eFigure 5 and eTable 6 in Supplement 1).

#### Subgroup and Sensitivity Analyses

We repeated our analyses within 4 subgroups: female enrollees with an intellectual or developmental disability (IDD), physical disability, sensory disability, or mental health–related disability. These categories are not mutually exclusive or encompassing of the entire sample. We identified groups using Chronic Conditions Warehouse codes (created using condition-specific enrollment periods) and *ICD-10* codes listed in eTable 7 in Supplement 1.^25^

We conducted sensitivity analyses to test the robustness of our findings to alternative time periods, samples, and model specifications (eMethods 5 in Supplement 1). We removed months during the COVID-19 public health emergency, used available claims from 2010 to 2015 to identify receipt of contraceptive methods that would remain effective during the study period, and restricted the sample to those with and without medical conditions treated using contraceptives (eg, endometriosis), as additional coverage is available for medically necessary contraception. We also tested using covariate adjustment instead of propensity score weighting, using later-treated rather than never-treated units as the sDID control group, running a balanced panel, and using a 2-way fixed-effects model that did not account for staggered treatment timing.

We then evaluated switching behavior for contraceptive methods and performed 2 placebo tests to provide evidence that observed changes in contraceptive use following a transition from Medicare to dual enrollment resulted from gaining contraceptive coverage rather than experiencing any insurance change. Specifically, we examined the association of 2 alternative transitions (dual enrollment to Medicare and Medicaid to dual enrollment) with contraceptive use and the association of a transition from Medicare to dual enrollment with use of a preventive reproductive health service (eg, Papanicolaou test) that is fully covered by each insurance type.

## Results

Our sample included 51 501 303 monthly observations from 1 606 129 women with disabilities. The mean (SD) age at observations was 35.93 (8.58) years. Across all insurance types, 1.8% of monthly observations were from Asian women, 30.7% from Black women, 13.0% from Hispanic women, 52.6% from White women, and 1.9% from multiracial women or women identifying as another race and ethnicity. Before propensity score weighting, those enrolled in TM and MA were older compared with those dual enrolled or enrolled in Medicaid, more often non-Hispanic White, and more likely to reside in zip codes with higher median incomes, lower poverty rates, and higher rates of attainment of 4-year college degrees (Table 1). Across all 5 insurance types, mental health–related disabilities were the most common, followed by physical disabilities, IDDs, and sensory disabilities.

**Table 1.** Characteristics of National Sample of Reproductive-Aged Female Enrollees With Social Security Disability Insurance– or Supplemental Security Income–Eligible Disabilities by Public Insurance Type and Insurance Transition

Characteristic	Contraceptive use by public insurance exposure group					Association of gaining coverage with contraceptive use	
	TM alone	MA alone	Dual TM-Medicaid	Dual MA-Medicaid	Medicaid alone	Continuous Medicare^a^	Transition to dual enrollment^b^
No. of monthly observations	3 843 679	1 047 134	5 056 059	1 806 931	39 747 500	1 540 812	803 123
No. of individuals	164 779	45 375	169 214	70 763	1 335 359	71 096	39 387
Age group, %							
20-24 y	2.7	1.0	4.6	2.5	14.5	1.4	8.7
25-29 y	4.7	2.5	10.0	7.7	16.3	3.3	9.6
30-34 y	9.8	6.1	15.7	13.9	16.8	8.0	14.3
35-39 y	17.1	13.8	20.2	19.8	16.3	15.9	19.2
40-44 y	25.7	26.7	22.5	24.2	16.2	27.2	21.7
45-49 y	40.1	49.9	27.1	31.9	19.9	44.1	26.6
Race and ethnicity, %^c^							
Asian	2.0	1.5	1.9	2.0	2.0	1.9	1.9
Black	17.4	15.7	23.5	30.9	31.6	15.2	25.8
Hispanic	6.5	11.0	6.8	10.7	15.2	7.4	8.2
White	72.2	69.9	65.3	54.6	49.5	73.5	61.8
Multiracial or other^d^	2.0	2.0	2.6	1.9	1.7	2.0	2.3
County-level MA coverage, mean (SD), %	34.0 (14.3)	42.3 (16.5)	33.6 (14.5)	41.3 (12.1)	29.1 (11.9)	36.8 (15.7)	33.3 (13.8)
Zip code–level characteristics, mean (SD)							
Median household income, $ US	56 090.4 (21 682.0)	51 254.5 (22 205.4)	51 897.6 (19 778.7)	48 699.1 (18 563.0)	47 690.1 (18 389.3)	55 701.0 (22 277.1)	51 858.6 (19 877.2)
Living in poverty, %	15.8 (9.6)	19.2 (13.2)	17.8 (9.7)	20.1 (10.5)	20.7 (10.5)	16.2 (10.7)	18.0 (9.9)
4-y College degree, %	26.8 (14.4)	25.9 (13.6)	25.0 (13.8)	23.5 (13.2)	22.4 (12.9)	27.0 (14.3)	25.0 (13.7)
Disability type, %							
Intellectual or developmental	5.9	8.7	20.7	17.0	11.2	6.0	13.5
Physical	24.6	29.1	34.4	35.0	16.5	24.6	35.9
Sensory	5.1	7.1	11.4	10.6	6.6	5.4	7.5
Mental health related	60.3	78.0	81.8	83.9	71.3	62.7	80.9

Abbreviations: MA, Medicare Advantage; TM, traditional Medicare.

^a^ Control group with TM or MA coverage.

^b^ Includes dual TM-Medicaid and dual MA-Medicaid.

^c^ Determined using the Medicare Minimum Beneficiary Summary File and Medicaid Transformed Analytic Files Demographic and Eligibility file provided by the Centers for Medicare & Medicaid Services.

^d^ Includes racial and ethnic groups other than Asian, Black, Hispanic, or White.

### Contraceptive Use by Insurance Type

**Figure 1.**
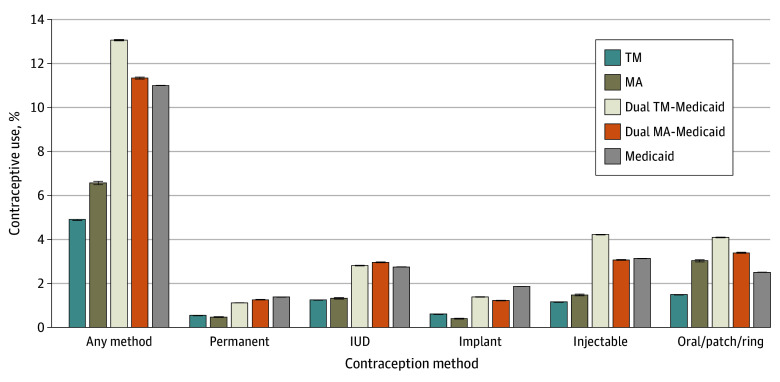
Contraceptive Use by Public Insurance Type The estimated probabilities of contraceptive use are shown among women with disabilities enrolled in traditional Medicare (TM), Medicare Advantage (MA), dual TM-Medicaid, dual MA-Medicaid, and Medicaid. The bars represent 95% CIs. IUD indicates intrauterine device.

The estimated probability of use of any contraceptive method was lowest among women enrolled in TM (4.9%; 95% CI, 4.9%-4.9%) and MA (6.6%; 95% CI, 6.5%-6.6%), followed by women enrolled in Medicaid (11.0%; 95% CI, 11.0%-11.0%), dual MA-Medicaid (11.3%; 95% CI, 11.3%-11.4%), and dual TM-Medicaid (13.1%; 95% CI, 13.0%-13.1%) (*P* < .01) (Figure 1). This trend of lower use among women enrolled in Medicare alone compared with women enrolled in Medicaid and dual enrolled held for each contraceptive method except oral contraceptives, patches, and rings, which had a higher estimated probability of use among women enrolled in MA (3.0%; 95% CI, 3.0%-3.1%) compared with those enrolled in Medicaid (2.5%; 95% CI, 2.5%-2.5%).

### Association of Gaining Coverage With Contraceptive Use

Our subsample included 2 343 935 monthly observations from 110 483 female enrollees (39 387 treatment, 71 096 control). In the 12 months prior to a transition from Medicare to dual enrollment, the estimated probability of any contraceptive use was 8.6% (95% CI, 8.5%-8.7%). An estimated 1.8% (95% CI, 1.8%-1.8%) were using permanent methods, 2.8% (95% CI, 2.7%-2.8%) were using long-acting methods, and 4.2% (95% CI, 4.1%-4.3%) were using short-acting methods (Table 2). In the 12 months following a transition from Medicare alone to dual enrollment, the estimated probability of use of any method increased to 11.9% (95% CI, 11.8%-12.0%). An estimated 2.3% (95% CI, 2.3%-2.4%) were using permanent methods, 3.9% (95% CI, 3.9%-4.0%) were using long-acting methods, and 6.0% (95% CI, 5.9%-6.0%) were using short-acting methods during this period.

**Table 2.** Association of Gaining Contraceptive Coverage Through a Transition From Medicare to Dual Medicare-Medicaid Enrollment With Contraceptive Use^a^

Contraceptive method	Pretransition use, % (95% CI)		Posttransition use, % (95% CI)		Point change, % (95% CI)	
	12-mo Mean	1 mo Before transition	12-mo Mean	12 mo After transition	12-mo Mean	12 mo After transition
Any	8.6 (8.5-8.7)	9.9 (9.6-10.2)	11.9 (11.8-12.0)	14.1 (13.7-14.6)	2.7 (2.5-2.9)	3.9 (3.5-4.3)
Permanent	1.8 (1.8-1.8)	2.0 (1.9-2.2)	2.3 (2.3-2.4)	2.6 (2.4-2.8)	0.1 (0.0-0.1)	0.1 (0.1-0.2)
Long-acting	2.8 (2.7-2.8)	3.4 (3.2-3.6)	3.9 (3.9-4.0)	4.9 (4.6-5.2)	0.8 (0.7-0.9)	1.4 (1.2-1.6)
IUD	2.0 (1.9-2.0)	2.3 (2.2-2.5)	2.9 (2.8-2.9)	3.5 (3.3-3.7)	0.5 (0.4-0.6)	0.8 (0.7-1.0)
Implant	0.9 (0.9-0.9)	1.2 (1.0-1.3)	1.2 (1.2-1.2)	1.5 (1.4-1.7)	0.3 (0.3-0.4)	0.6 (0.5-0.7)
Short-acting	4.2 (4.1-4.3)	4.8 (4.6-5.1)	6.0 (5.9-6.0)	7.1 (6.8-7.4)	1.9 (1.7-2.2)	2.6 (2.3-3.0)
Injectable	2.1 (2.0-2.1)	2.5 (2.3-2.6)	2.7 (2.7-2.8)	3.4 (3.1-3.6)	0.7 (0.6-0.9)	1.0 (0.8-1.3)
Oral, patch, or ring	2.2 (2.1-2.2)	2.4 (2.3-2.6)	3.3 (3.2-3.3)	3.8 (3.6-4.0)	1.2 (1.1-1.4)	1.6 (1.3-1.9)

Abbreviation: IUD, intrauterine device.

^a^ Percentages represent propensity score–weighted estimated probabilities of contraceptive use.

**Figure 2.**
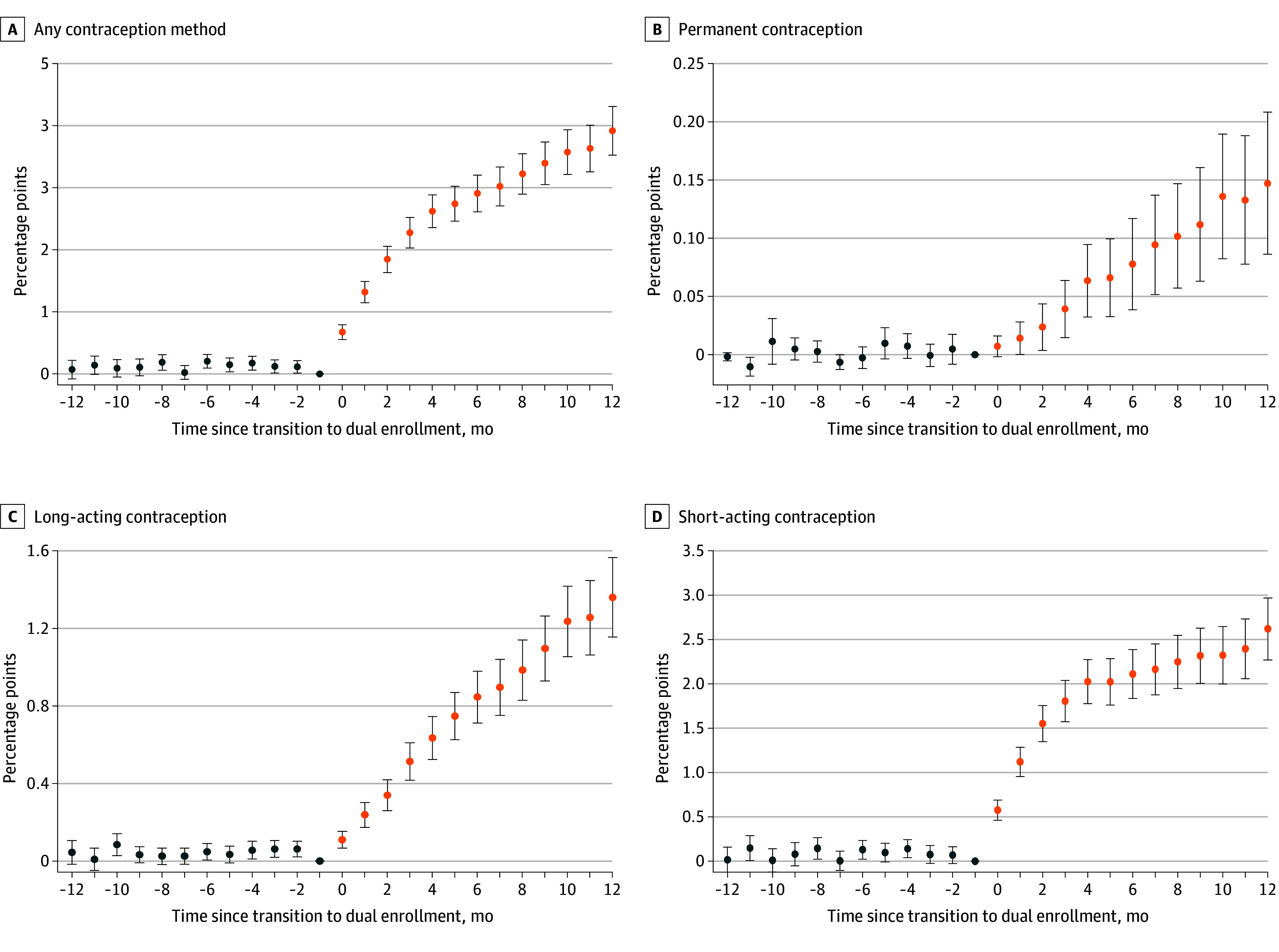
Association of Gaining Contraceptive Coverage Through a Transition From Medicare to Dual Medicare-Medicaid Enrollment With Contraceptive Use The percentage point changes in the estimated probabilities of any contraceptive use (A), use of a permanent method (B), use of a long-acting method (C), and use of a short-acting method (D) are shown before and after a transition from Medicare alone to dual Medicare-Medicaid enrollment. Values are from propensity score–weighted staggered difference-in-differences models with estimators from Callaway and Sant’Anna.^21^ The bars represent 95% CIs. Blue dots represent pretransition associations and orange dots represent posttransition associations.

Gaining contraceptive coverage through dual enrollment was associated with an increase of 3.9 (95% CI, 3.5-4.3) percentage points (35%) in use of any contraceptive method by the 12th month after a transition, corresponding to an additional 13 523 women with disabilities nationally using any contraceptive method. This included an increase of 0.1 (95% CI, 0.1-0.2) percentage points (30%) in the use of permanent methods, 1.4 (95% CI, 1.2-1.6) percentage points (44%) in the use of long-acting methods, and 2.6 (95% CI, 2.3-3.0) percentage points (45%) in the use of short-acting methods (Figure 2).

### Subgroup and Sensitivity Analyses

Overall, patterns in contraceptive use by insurance type were similar within each of the disability subgroups. The probability of any contraceptive use was slightly higher among women with IDDs and sensory disabilities with each insurance type compared with all women and women with physical and mental health–related disabilities (eFigure 6 in Supplement 1). The association of gaining contraceptive coverage with contraceptive use was greater among women with IDDs at 6.1 (95% CI, 4.7-7.5) percentage points by month 12 compared with all women but similar among women with physical, sensory, and mental health–related disabilities (eTable 8 in Supplement 1).

Our results were robust to changes in the study period, sample, and model specifications (eTables 9-16 and eFigures 7-11 in Supplement 1). Notably, the association of gaining contraceptive coverage with any contraceptive use increased by 4.3 (95% CI, 3.8-4.7) percentage points after removing months affected by the COVID-19 public health emergency. Gaining contraceptive coverage led to an increase of 1.0 (95% CI, 0.3-1.8) percentage points in switching from a short-acting to a long-acting or a permanent contraceptive method by month 12 (eTable 17 in Supplement 1). Placebo tests increased our confidence that observed increases in contraceptive use were associated with increased contraceptive coverage rather than experiencing any insurance change (eTables 18-20 and eFigure 12 in Supplement 1).

## Discussion

In this national cross-sectional study of contraceptive use in the Medicare program among women with SSDI- and/or SSI-eligible disabilities, we have 2 key findings. First, women with disabilities enrolled in TM and MA who are subject to out-of-pocket costs for contraceptive methods had lower contraceptive use compared with those enrolled in Medicaid or dual enrolled who are not subject to those costs. Second, gaining coverage of contraceptives through a transition from Medicare to dual enrollment was associated with an increase of 3.9 percentage points in the use of any contraceptive method, equating to nearly 14 000 more women with disabilities using any contraceptive method or a 35% increase in use. This increase is notable when considered alongside the 32% gap in contraceptive use between US women with and without disabilities.^1,2^ These findings suggest that Medicare’s coverage policies may pose a financial barrier to desired contraceptive use.

Medicare is the only form of US health insurance in which the entire population of reproductive age is disabled. By offering less generous contraceptive coverage than other health insurance payers, the program perpetuates and potentially exacerbates disability-related disparities in contraceptive use. In recognition of this inequity, in June 2023, the Department of Health and Human Services updated the 2024 Medicare Part D formulary clinical review to include implants and IUDs for the first time in the program’s history.^26^ While this change was an important step toward improving contraceptive access for Medicare enrollees, as of December 2024, no plans to cover permanent methods or eliminate cost-sharing have been announced.

Based on our findings, we recommend the following actions to capitalize on policy momentum around Medicare coverage of contraceptives. First, we recommend that the Centers for Medicare & Medicaid Services use rulemaking to require Medicare to cover prescription contraceptive methods without cost-sharing. We found that when Medicare enrollees were no longer subject to cost-sharing for short- or long-acting methods, their use of these methods increased. Out-of-pocket costs for contraceptives vary widely, with reported costs for IUDs ranging from $500 to $14 000 ($100-$2800 with 20% coinsurance).^27,28^ Our results suggest that any amount of cost-sharing for contraceptives, even for low-cost methods such as oral contraceptives, may be prohibitive for this population of women receiving SSDI benefits (a mean of $1226 per month in 2023 for female enrollees aged 18-49 years).^29^ Second, we recommend that Congress pass a statutory amendment to require Medicare to cover desired permanent contraceptive methods for pregnancy prevention. We found that gaining coverage of permanent methods led to a small but significant increase in use among Medicare enrollees.

It is crucial that these policies be accompanied by actions to increase access to person-centered contraceptive counseling and reproductive autonomy. In 2025, 30 states and Washington, DC, still maintained laws allowing for nonconsensual sterilization of disabled people, and in 2019, Iowa and Nevada unanimously passed laws enabling forced sterilization of disabled people under guardianship.^30,31^ In health care settings, women with disabilities continue to encounter discomfort from clinicians when discussing their sexuality and pressure to accept permanent or long-acting contraceptives.^32^ Repealing forced contraception laws and training clinicians in antiableist care and shared contraceptive decision-making^33^ are necessary steps toward a future where people with disabilities can choose contraceptive methods that align with their reproductive goals.

### Limitations

This study has some limitations. Medicare and Medicaid claims do not record contraceptives provided at free clinics, paid for entirely out of pocket without a corresponding insurance claim, or received prior to the study period. In addition, claims do not contain information on contraceptive desires, so we cannot restrict our sample to women seeking contraceptives for pregnancy prevention. We also cannot know whether a woman changed her contraceptive use in response to a life event that also changed her Medicaid eligibility, such as a change in household income through marriage.

## Conclusions

In this cross-sectional study of contraceptive use in the Medicare program, we found evidence that Medicare’s contraceptive coverage gaps were associated with restricted contraceptive use among disabled enrollees. Based on our findings, we recommend that Congress require Medicare to cover all US Food and Drug Administration–approved contraceptive methods without cost-sharing. Doing so would reduce financial barriers to desired contraceptives and align Medicare’s coverage requirements with those of Medicaid, private insurance plans, and TRICARE.

## Notes

Supplement 1. **eMethods 1.** Sample Construction **eFigure 1.** Flowchart of Study Inclusion **eMethods 2.** Diagnosis, Procedure, and Drug Codes **eTable 1.** Diagnosis and Procedure Codes for Contraceptives and Contraceptive Exclusions **eTable 2.** National Drug Codes **eMethods 3.** Propensity Scores **eTable 3.** Propensity Scores Summary **eFigure 2.** Visual Test of Common Support for Analysis 1 (Left) and Analysis 2 (Right) Propensity Score Models **eTable 4.** Characteristics of Groups Before and After Propensity Score Weighting **eFigure 3.** Standardized Mean Differences for Analysis 1 Before (A) and After (B) Propensity Score Weighting and Analysis 2 Before (C) and After (D) Propensity Score Weighting **eTable 5.** Characteristics of Propensity Score Outliers **eFigure 4.** Crude Contraceptive Method Use by Public Insurance Type **eMethods 4.** Staggered Difference-in-Differences Model **eFigure 5.** Crude Contraceptive Use Before and After a Real or Proxy Transition in Insurance Coverage **eTable 6.** Logistic Regression of Association Between Contraceptive Method Use and Month in the Prestudy Period, Comparing the Treatment and Control Groups **eTable 7.** Disability Type Codes **eFigure 6.** Propensity Score–Weighted Contraceptive Use by Public Insurance and Disability Type **eTable 8.** sDID Results by Disability Type **eMethods 5.** Sensitivity Analyses **eFigure 7.** Estimated Probability of Contraceptive Use, January 2016 to February 2020 **eTable 9.** sDID results, January 2016 to February 2020 **eFigure 8.** Estimated Probability of Contraceptive Use Within the Main Analysis Sample Between 2016 and 2020, Including Any Available 2010-2015 Look-Back Data **eTable 10.** sDID Results Including Look-Back Data **eTable 11.** Codes for Clinical Indications for Contraceptives **eFigure 9.** Estimated Probability of Contraceptive Among Women With a Clinical Indication for Contraception Use **eFigure 10.** Estimated Probability of Contraceptive Use Among Women Without a Clinical Indication for Contraception Use **eTable 12.** sDID Results Among Women With and Without a Clinical Indication for Contraceptive Use **eFigure 11.** Covariate-Adjusted Contraceptive Use **eTable 13.** sDID Results Using Covariate Adjustment **eTable 14.** sDID Results Using Later Transition as Control Group **eTable 15.** sDID Results Using Balanced sDID Model **eTable 16.** Two-Way Fixed-Effects Model **eTable 17.** Association of a Transition From Medicare to Dual Enrollment on Contraceptive Method Switching Among Women Using a Contraceptive Method **eTable 18.** sDID Results Showing Association of Alternative Transitions With Contraceptive Use **eFigure 12.** Event Study Plots of Contraceptive Use After Dual Enrollment to Medicare Transition (Left) and Medicaid to Dual Transition (Right), 2016 to 2020
eReferences.

Supplement 2.
Data Sharing Statement
