# Supplementary material for: Coverage Gaps and Contraceptive Use Among Medicare Enrollees With Disabilities
Source: JAMA Netw Open. 2025 Jun 25;8(6):e2517718. doi: 10.1001/jamanetworkopen.2025.17718 (PMC12199052; doi:10.1001/jamanetworkopen.2025.17718)
Supplement: Supplement 1. — eMethods 1. Sample Construction eFigure 1. Flowchart of Study Inclusion eMethods 2. Diagnosis, Procedure, and Drug Codes eTable 1. Diagnosis and Procedure Codes for Contraceptives and Contraceptive Exclusions eTable 2. National Drug Codes eMethods 3. Propensity Scores eTable 3. Propensity Scores Summary eFigure 2. Visual Test of Common Support for Analysis 1 (Left) and Analysis 2 (Right) Propensity Score Models eTable 4. Characteristics of Groups Before and After Propensity Score Weighting eFigure 3. Standardized Mean Differences for Analysis 1 Before (A) and After (B) Propensity Score Weighting and Analysis 2 Before (C) and After (D) Propensity Score Weighting eTable 5. Characteristics of Propensity Score Outliers eFigure 4. Crude Contraceptive Method Use by Public Insurance Type eMethods 4. Staggered Difference-in-Differences Model eFigure 5. Crude Contraceptive Use Before and After a Real or Proxy Transition in Insurance Coverage eTable 6. Logistic Regression of Association Between Contraceptive Method Use and Month in the Prestudy Period, Comparing the Treatment and Control Groups eTable 7. Disability Type Codes eFigure 6. Propensity Score–Weighted Contraceptive Use by Public Insurance and Disability Type eTable 8. sDID Results by Disability Type eMethods 5. Sensitivity Analyses eFigure 7. Estimated Probability of Contraceptive Use, January 2016 to February 2020 eTable 9. sDID results, January 2016 to February 2020 eFigure 8. Estimated Probability of Contraceptive Use Within the Main Analysis Sample Between 2016 and 2020, Including Any Available 2010-2015 Look-Back Data eTable 10. sDID Results Including Look-Back Data eTable 11. Codes for Clinical Indications for Contraceptives eFigure 9. Estimated Probability of Contraceptive Among Women With a Clinical Indication for Contraception Use eFigure 10. Estimated Probability of Contraceptive Use Among Women Without a Clinical Indication for Contraception Use eTable 12. sDID Results Among Women With and Without a Clin [file jamanetwopen-e2517718-s001.pdf]

Supplementary Online Content

Bellerose M, Ellison J, Steenland MW, Meyers DJ, Mitra M, Shireman TI. Coverage gaps and contraceptive use among Medicare enrollees with disabilities. *JAMA Netw Open.* 2025;8(6):e2517718. doi:10.1001/jamanetworkopen.2025.17718

eMethods 1. Sample Construction
eFigure 1. Flowchart of Study Inclusion
eMethods 2. Diagnosis, Procedure, and Drug Codes
eTable 1. Diagnosis and Procedure Codes for Contraceptives and Contraceptive Exclusions
eTable 2. National Drug Codes
eMethods 3. Propensity Scores
eTable 3. Propensity Scores Summary
eFigure 2. Visual Test of Common Support for Analysis 1 (Left) and Analysis 2 (Right) Propensity Score Models
eTable 4. Characteristics of Groups Before and After Propensity Score Weighting
eFigure 3. Standardized Mean Differences for Analysis 1 Before (A) and After (B) Propensity Score Weighting and Analysis 2 Before (C) and After (D) Propensity Score Weighting
eTable 5. Characteristics of Propensity Score Outliers
eFigure 4. Crude Contraceptive Method Use by Public Insurance Type
eMethods 4. Staggered Difference-in-Differences Model
eFigure 5. Crude Contraceptive Use Before and After a Real or Proxy Transition in Insurance Coverage
eTable 6. Logistic Regression of Association Between Contraceptive Method Use and Month in the Prestudy Period, Comparing the Treatment and Control Groups
eTable 7. Disability Type Codes
eFigure 6. Propensity Score–Weighted Contraceptive Use by Public Insurance and Disability Type
eTable 8. sDID Results by Disability Type
eMethods 5. Sensitivity Analyses
eFigure 7. Estimated Probability of Contraceptive Use, January 2016 to February 2020
eTable 9. sDID results, January 2016 to February 2020
eFigure 8. Estimated Probability of Contraceptive Use Within the Main Analysis Sample Between 2016 and 2020, Including Any Available 2010-2015 Look-Back Data
eTable 10. sDID Results Including Look-Back Data
eTable 11. Codes for Clinical Indications for Contraceptives
eFigure 9. Estimated Probability of Contraceptive Among Women With a Clinical Indication for Contraception Use
eFigure 10. Estimated Probability of Contraceptive Use Among Women Without a Clinical Indication for Contraception Use

© 2025 Bellerose M et al. *JAMA Network Open*.

eTable 12. sDID Results Among Women With and Without a Clinical Indication for Contraceptive Use
eFigure 11. Covariate-Adjusted Contraceptive Use
eTable 13. sDID Results Using Covariate Adjustment
eTable 14. sDID Results Using Later Transition as Control Group
eTable 15. sDID Results Using Balanced sDID Model
eTable 16. Two-Way Fixed-Effects Model
eTable 17. Association of a Transition From Medicare to Dual Enrollment on Contraceptive Method Switching Among Women Using a Contraceptive Method
eTable 18. sDID Results Showing Association of Alternative Transitions With Contraceptive Use
eFigure 12. Event Study Plots of Contraceptive Use After Dual Enrollment to Medicare Transition (Left) and Medicaid to Dual Transition (Right), 2016 to 2020
eReferences.

This supplementary material has been provided by the authors to give readers additional information about their work.

© 2025 Bellerose M et al. *JAMA Network Open*.

eMethods 1. Sample Construction

Datasets

This analysis used national 2016-2020 Traditional Medicare (TM), Medicare Advantage (MA), and Medicaid claims and corresponding enrollment files. We included TM claims from a 20% random sample of enrollees, MA encounter records from a 20% random sample of enrollees, and Medicaid claims from a 100% sample of enrollees aged 12 and older. To identify our sample, we used monthly enrollment and demographic information from the Medicare Beneficiary Summary File (MBSF) and Transformed Medicaid Statistical Information System (T-MSIS) Demographic and Eligibility (DE) files. To identify contraceptive use, we used inpatient, outpatient, physician, and pharmaceutical claims. Each of these datasets contain consistent person identifiers, allowing them to be linked longitudinally to construct a master dataset including monthly enrollment in TM, MA, and/or Medicaid.

Medicare and Medicaid eligibility

Medicare is available to people aged 65 and older, people with End State Renal Disease (ESRD) or Amyotrophic Lateral Sclerosis (ALS), and people with disabilities who have received Social Security Disability Insurance (SSDI) for two years. SSDI is a federal benefits program available to people aged 18 and older who cannot work due to disability. To qualify, a person (or their parent or spouse with additional restrictions) must have worked in a job covered by Social Security and meet the Social Security Administration's (SSA) definition of disability, which is "unable to engage in a substantial gainful activity because of a medically determined physical or mental impairment expected to last at least 12 months or until death." In 2022, the most common qualifying conditions among women were musculoskeletal system and connective tissue disorders (e.g., arthritis) at 32.5%, depressive, bipolar, and related disorders at 14.9%, nervous system and sensory organ issues (e.g., visual impairment) at 10.8%, and intellectual disorders at 8.0%.[1] At the time of enrollment, people can select TM or an MA plan, which may offer supplemental benefits but limit access to specific clinicians and desired specialty care.

Medicaid is available to adults with household incomes between 0-380% of the federal poverty level based on state eligibility levels, pregnancy, and parental status.[2] Additionally, states have flexibility to offer Medicaid to people with disabilities with incomes above these limits, including people enrolled in Supplemental Security Income (SSI).[3] Like SSDI, SSI is a federal benefits program run by the SSA. It provides monthly payments to disabled people with low household incomes. Receipt of SSI is not contingent upon work history. SSDI and SSI use the same set of disabling conditions for program eligibility.

People who meet the eligibility criteria for Medicare and Medicaid may dual enroll in both programs. Dual enrollees receive Medicaid coverage of Medicare premiums and reduced cost-sharing for a variety of services, supports, and medications. People whose household incomes are too high to qualify for full Medicaid may still qualify for support with Medicare premiums through enrollment in a Medicare Savings Program.[4] This is referred to as partial dual enrollment.

© 2025 Bellerose M et al. *JAMA Network Open*.

Sample inclusion

A flow diagram of study inclusion is shown in eFigure 1. We first identified females, ages 20-49, living in one of the 50 US states or the District of Columbia who were enrolled in TM, MA, or Medicaid for at least one month between 2016 to 2020 using the annual MBSF and DE files. We selected 20 as the minimum age for our sample because SSDI is available at age 18 and Medicare is available after two years of SSDI enrollment. During this stage, we removed people with ESRD and ASL from the Medicare sample as they are not subject to Medicare's disability eligibility criteria and waiting period. We then removed people who died within the study period.

Next, we restricted the Medicaid sample to people enrolled in SSDI or SSI in order to create Medicare and Medicaid samples including people with the same set of government recognized disabilities. The Medicaid analytic files for our sample had complete information on monthly SSDI and SSI receipt for over 80% of enrollees in line with Data Quality (DQ) Atlas reports.[5]

We then linked the MBSF and DE files using person identifiers. We removed months when individuals were not enrolled in TM, MA, or Medicaid and removed people who were partially dual enrolled. In order to restrict our sample to those most likely to be eligible for contraception, we also removed months during or following a claim for hysterectomy (i.e. surgical removal of the uterus or womb) or pregnancy based on procedure codes, as well as the 10 months prior to childbirth hospitalization. Finally, we removed observations missing age category, race/ethnicity, state, county, or zip code.

Identifying monthly insurance type

We identified monthly insurance type (TM alone, MA alone, dual TM-Medicaid, dual MA-Medicaid, Medicaid alone) using the monthly MA enrollment variable from the MBSF file and the monthly dual enrollment variables from the MBSF and DE files. We found strong overlap between the dual enrollment variables at the point of data linkage; 97.8% of women identified as full dual in the MBSF file in a given month were identified as full dual in that month in the DE file. When one file had a missing value for the dual enrollment variable, but the other file had a value for the dual enrollment variable, we used the complete record. When both files had a complete record, but there was disagreement, we used information from the MBSF, as Medicare is the primary payer for contraceptive care.

© 2025 Bellerose M et al. *JAMA Network Open*.

eFigure 1. Flowchart of Study Inclusion

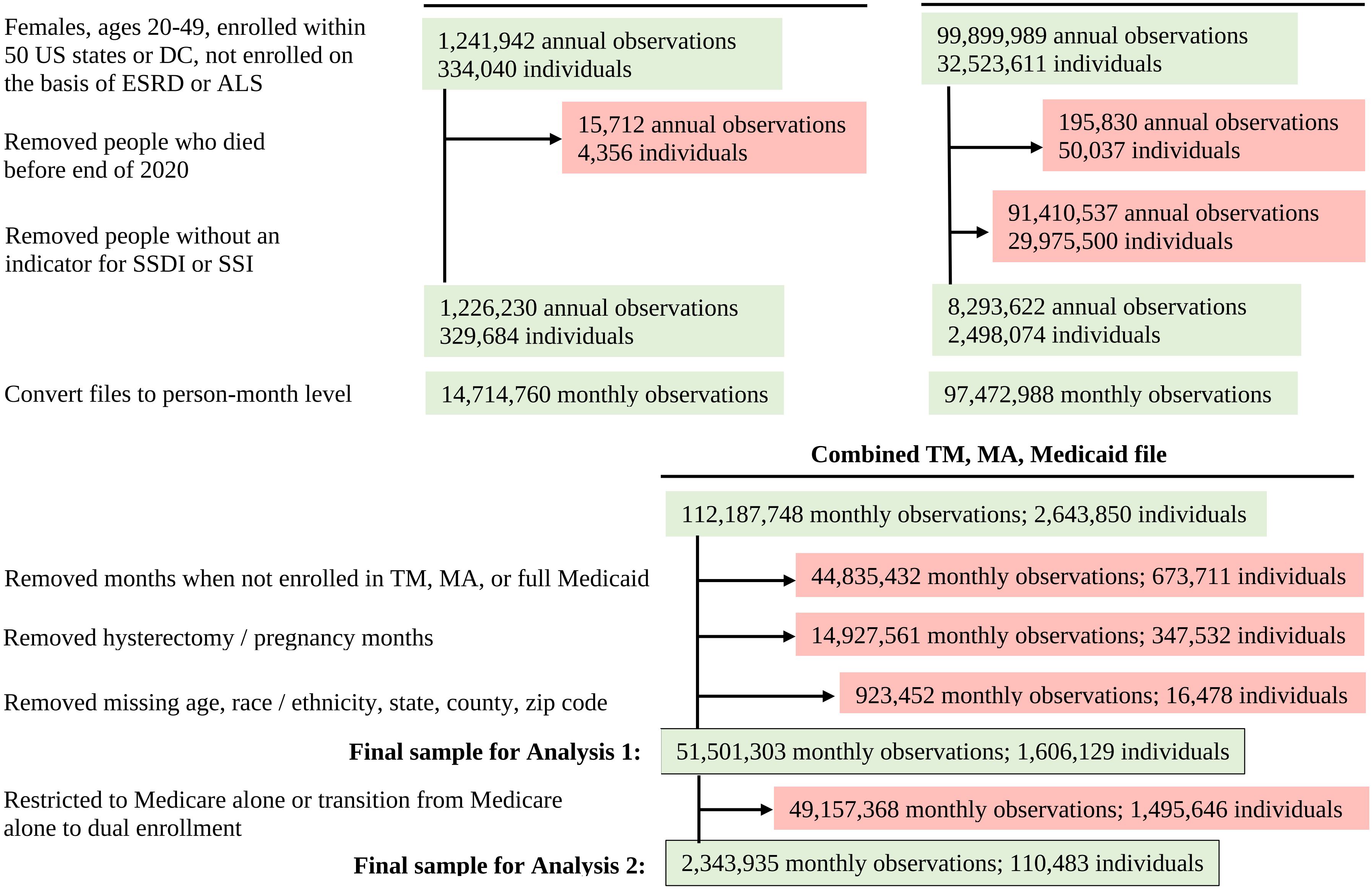

© 2025 Bellerose M et al. *JAMA Network Open*.

eMethods 2. Diagnosis, Procedure, and Drug Codes

In eTable 1, we list the diagnosis and procedure codes used to identify inpatient and outpatient claims for tubal ligation, salpingectomy, IUDs, implants, injectables, patches or rings, and oral contraceptives, as well as exclusion codes for childbirth, pregnancy, and hysterectomy. In eTable 2 we list the National Drug Codes used to identify pharmaceutical claims for IUDs, implants, injectables, patches or rings, and oral contraceptives.

eTable 1. Diagnosis and Procedure Codes for Contraceptives and Contraceptive Exclusions

	CPT codes	DRG codes	HCPCS codes	ICD-9 diagnosis codes	ICD-9 procedure codes	ICD-10 diagnosis codes	ICD-10 procedure codes
Tubal ligation / salpingectomy	58700		A4264	V5042<br>V252T<br>6282T<br>9989T	6621<br>6622<br>6629<br>6631<br>6632<br>6639<br>6640<br>6651<br>6652<br>6661<br>6662<br>6663<br>6692<br>6697	Z302	0U574ZZ<br>0U578ZZ<br>0UL74CZ<br>0UL74DZ<br>0UL74ZZ<br>0UL78DZ<br>0UL78ZZ<br>58565<br>58600<br>58605<br>58611<br>58615<br>58670<br>58671<br>A4264<br>0U570ZZ<br>0U573ZZ<br>0U577ZZ<br>0UL70CZ<br>0UL70DZ<br>0UL70ZZ<br>0UL73CZ<br>0UL73DZ<br>0UL73ZZ<br>0UL77DZ<br>0UL77ZZ<br>0UT70ZZ

© 2025 Bellerose M et al. *JAMA Network Open*.

							0UT74ZZ<br>0UT77ZZ<br>0UT78ZZ<br>0UT7FZZ<br>0567T
IUD insertion	J7300<br>58301<br>58300<br>J7302		58300<br>Q0090<br>J7296<br>J7297<br>J7298<br>J7300<br>J7301<br>J7302<br>J7303<br>S4981<br>S4989	V4552<br>V2542<br>V2512<br>V2511<br>99632<br>V2513<br>V2511	69.7	Z30014<br>Z30430<br>Z30431<br>Z30433<br>T8331XA<br>T8331XD<br>T8331XS<br>T8332XA<br>T8332XD<br>T8332XS<br>T8339AD<br>T8339XA<br>T8339XD<br>T8339XS	
IUD removal						Z30432	58301
Implant insertion	J7307<br>11982<br>11981<br>11983<br>11975			V2551<br>V2543<br>V4552		Z30017<br>Z3046	11981<br>11983<br>J7306<br>J7307<br>0JHD0HZ<br>0JHD3HZ<br>0JHF0HZ<br>0JHF3HZ<br>0JHG0HZ<br>0JHG3HZ<br>0JHH0HZ<br>0JHH3HZ
Implant removal			J7306<br>J7307<br>11981<br>11983				11982
Injectable	J1055		J1050<br>J1055			Z30013<br>Z3042	
Patch / ring			J7304			Z30016	

© 2025 Bellerose M et al. *JAMA Network Open*.

			J7303<br>J7304<br>J7294<br>J7295			Z3045<br>Z30015<br>Z3044	
Contraceptive pill	S4993<br>J7303<br>J7304		S4993			Z30011<br>Z3041	
Exclusions							
Childbirth hospitalization						O80<br>O82<br>Z37<br>10E0XZZ<br>10D00Z0<br>10D00Z1<br>10D00Z2<br>10D07Z3<br>10D08Z4<br>10D08Z5<br>10D08Z6<br>10D08Z7<br>10D07Z8<br>0196*<br>99464<br>99436<br>59622	
Pregnancy		765-770<br>774 -788<br>796-798<br>805-807<br>817- 819<br>831-83<br>998				O*<br>Z33<br>Z34<br>Z36<br>Z37<br>Z38<br>Z39<br>Z3A<br>10A	0U950ZZ<br>0U960ZZ<br>0U970ZZ<br>0UC50ZZ<br>0UC53ZZ<br>0UC54ZZ<br>0UC57ZZ<br>0UC58ZZ<br>0UC60ZZ<br>0UC63ZZ<br>0UC64ZZ<br>0UC67ZZ<br>0UC68ZZ<br>0UC70ZZ<br>0UC73ZZ

© 2025 Bellerose M et al. *JAMA Network Open*.

							0UC74ZZ<br>0UC77ZZ<br>0UC78ZZ<br>0UP8<br>0UW8<br>0UT5FZZ<br>0UT60ZZ<br>0UT64ZZ<br>0UT67ZZ<br>0UT68ZZ<br>0UT6FZZ<br>0UT78ZZ<br>0UT7FZZ<br>0UT50ZZ<br>0UT54ZZ<br>0UT57ZZ<br>0UT58ZZ<br>0U9780Z<br>0U9770Z<br>0U9740Z<br>0U9570Z<br>0U9580Z<br>0U9600Z<br>0U9640Z<br>0U9670Z<br>0U9700Z<br>0U9680Z<br>0UH80YZ<br>0UH83YZ<br>0UH84YZ<br>0U9500Z<br>0U9540Z
Hysterectomy	5815X<br>58180<br>582.00<br>58210<br>582.40<br>5826x<br>5827x<br>5828x			V880.1	683<br>6831<br>684<br>6851<br>6859<br>687<br>688<br>689		

© 2025 Bellerose M et al. *JAMA Network Open*.

					5829x<br>5854x<br>5855x<br>58552<br>5857x		

eTable 2. National Drug Codes

IUD	59365512801<br>50419042101	50419042308<br>50419042271	50419042408<br>23585801	52544003554<br>50419042471	51285020401<br>50419042208	51285020402<br>50419042201	50419042301<br>50419042401
Implant	52027201	52027401	52027480	52433001			
Injectable	50102059140<br>68788923301<br>54868410000<br>67457088799<br>54569561600<br>247210801<br>54569621900<br>16714098101<br>548541000	703680101<br>50090088300<br>52125091501<br>50090066500<br>54868334801<br>62756009040<br>59762453802<br>9062601<br>59762453701	9074630<br>62756009045<br>68071466605<br>69097053931<br>67457088700<br>67457088701<br>50090425100<br>548540000<br>9470913	9737607<br>703680104<br>50090332800<br>54868361300<br>71205011801<br>54569370100<br>9074635<br>23490585401	68071181805<br>54569552700<br>9737611<br>59762453702<br>9470901<br>54868410001<br>548541025<br>9737604	70518193200<br>548570100<br>548571100<br>16714099901<br>62756009140<br>54569490400<br>548540025<br>703681121	54868525700<br>16714098102<br>55045350501<br>548540125<br>59762453809<br>52125064001<br>59762453801<br>50090045900
Patch / ring	54569541300<br>50458019224<br>50458019215<br>378334053<br>52027305	76413013103<br>35356041003<br>54868483200<br>66993060581<br>66993060536	54569586500<br>55887075401<br>12822031301<br>52027301<br>78206014601	378334016<br>62192001<br>50458019201<br>62192015<br>378334032	52027381<br>52027304<br>50261031301<br>65162046932<br>78206014603	50090561100<br>52027303<br>62192024<br>54868467000<br>50090100800	378334017<br>52027385<br>54868483201<br>65162046935<br>50090168300

© 2025 Bellerose M et al. *JAMA Network Open*.

Contraceptive pill	31722093431	68180086011	68180086573	555071558	65862077828	51862029201	66993061528
	65862092687	54569662900	54569067900	51285008297	378729353	52544095121	70518132500
	68788723902	52544024941	52544025988	54569654200	52544016731	16714007302	62171415
	247151628	62178115	68180086012	378728098	51862026006	247223528	247198628
	65862084892	51862047106	54569143900	76413012828	68180087511	555901858	555903400
	70700015284	68180089371	54569422201	52544022829	53002171103	62179600	378728085
	52544026528	68180089213		247052028	52544055028	52544008741	69238103107
	430053514	50090341300	50419040201	555902658	76413013028	51285012079	54569662200
	68180089111	555912366	50419043312	378730053	65862092688	781565615	430053750
	52544017572	68180087573	603754001	68180089211	53002156706	53002156606	62125100
	51862000706	93603191	53002164701	430042095	254203280	68788683202	53002172201
	54569068501	68180087913	21695085601	68462072029	54868627600	378728485	50102030001
	65862077885	68788742902	16714036003	52028308	378730785	50458025106	52544094028
	68462071929	53002164606	50458019423	603752549	555906458	68462030584	59746076343
	68462039429	68180086511	53002177503	430057060	50458025115	60505418303	8253505
	93807328	53002156406	50458019416	62141123	53002163201	54569534300	50090147700
	51862003601	52544095021	50458019615	65862086495	430053595	51862028403	62125120
	555903458	62176120	68180084013	60219155406	68180089113	53002159101	53002177506
	16714034601	50458019411	70700015285	781557515	54868627400	16714036303	53002156603
	16714046402	50458019106	54868042800	65862077892	50458019400	68462050479	55045378106
	51862086606	54868436900	51285007997	16714034003	254203291	68180084473	555913167
	50419041112	16714036001	51285008070	59746076443	54569549300	53002156506	68462063784
	93209028	16714034701	93330528	16714034602	247176521	603752117	603762549
	55887005228	52544098228	603359049	61786038506	50458019712	51862051001	68180087311
	70700011485	53002164601	51285008370	51862089601	50102022021	555902058	51862064803
	555906467	17478026106	555903270	16714041303	16714036004	65862077685	68180089813
	555906558	51862064806	51862087203	52544095428	59651002928	378655085	53002157703
	70518084200	50419048303	57297087613	51862004791	603764201	52544025428	68180090471
	54569657500	254203080	54569068901	65862088788	603760615	52544029528	247201228
	16714040502	68462063729	51285011458	54569603200	68180084813	50419040701	51862054501
	65862077688	51862087206	50419040301	52544024828	50102010001	69238155106	53002164803
	68462064691	50090396200	51862064802	781558436	430053014	68180084313	53217009128
	21695077001	54868423900	50102025421	68180087711	16714044103	54569662400	68180084411
	54569628000	51862004501	53002145401	68180089413	50102025423	51862029206	555906658
	54868474200	53002160006	50458017115	50458019406	70700011684	54569664100	642747103
	781558315	50102022423	65862089788	50419040901	54569511500	70518121200	60505418301
	254203273	57297084413	68462055684	50102012801	68180087373	781558491	16714041604
	55045378302	51862087201	50419048301	378728885	68462931884	70700012084	247201008
	65862089992	54868485000	603766517	16714037002	51862086601	68180086571	50419048371
	51285043182	31722094531	378728398	68462056584	247198621	54569644200	50102012001

© 2025 Bellerose M et al. *JAMA Network Open*.

	50102023023	54868270100	50102030011	53002157701	430058514	603762517	76413010428
	54569657600	68462073384	50090138000	16714040704	68180087613	68180083711	50452025115
	378728585	16714036504	781411015	65862089988	378728490	51285008498	50419048372
	68462071984	16714037004	52544024531	430048295	51862023803	603763449	555901058
	70700012387	52544038428	52544024728	378728285	50102024021	52544025928	93330428
	65862088728	51862089401	68462056529	16714044001	51862089602	54868051600	51862026001
	52544095328	254202991	247139828	53002156501	75834013029	52544005441	58016482701
	21695099528	65862077787	53002162806	53002154901	54868151200	16714040603	50458017100
	66993061128	51862089603	16714044002	555904979	75854060203	16714037001	65862094087
	50102012048	54569645700	65862093521	603359017	65862089792	93532828	68180085773
	61786038206	8111720	52544038431	68180090311	65862077692	247214728	35356001568
	603763417	54868386300	65862092528	65862088692	68180087671	16714036703	54868404500
	68180090473	53002118803	68462055629	16714046403	430054050	50419041128	51862001206
	53002159103	68180090211	52544027428	16714046404	51862003603	76413011128	53002163103
	50090255900	50102030013	23490767001	52544098131	50458019120	52544029241	555906479
	52544005431	50419040771	68180089911	70700012185	57297088013	62133220	8251402
	54868616100	378729653	62171400	21695085501	68180091111	35356037028	68180090213
	50090323500	31722093428	50458019715	21695085701	430057014	51862089406	378730853
	430054014	68180089173	52544029128	62191015	68180086473	59651003028	68462030529
	54569422200	70700010184	50458019112	54868582800	378727256	603761049	54569579600
	53002163106	53002173203	254202980	378728590	430053950	51862051006	57297088211
	603760748	53002119106	51862056406	62191000	21695068528	68180086513	68462031829
	53002156601	603752149	52544038328	378655053	35356036828	50419040503	51862088403
	378728153	68462030929	52544005872	54569427301	59746076442	53002173401	50090143100
	54868050801	52544098231	54868482800	65862086583	60505418300	69238153106	53002164603
	555905179	93214028	555901467	55045349801	50458019428	50102013001	16714036302
	16714036503	68462065690	51285009287	52544055031	70700011985	57297085713	555906679
	16714034001	50102010003	52544062928	51862064501	555906667	52959045002	51285043187
	54868260600	68180084471	52544063028	16714034702	62190320	52544027536	52026108
	555904579	603751201	247201328	54868475400	430058045	53002145406	93313491
	53002168706	16714041603	50458025100	781405815	65862092587	555901279	59651003087
	51862007206	71205028728	65162034784	16714040403	68180088613	16714036603	68180091173
	62176100	75834012984	247151328	53002156503	52544027928	54878727500	54569489000
	52544089228	378729985	555902557	54569068500	57994000890	51862004701	54569498400
	53002160003	54569628101	53002163701	378729256	50458025112	35356036530	50090219100
	71205014428	57297084313	52544095931	52544023531	378727253	68180087673	16714040503
	35356002168	68180085713	50090147100	54868532600	16714034004	68462050481	53002164806
	555901458	53002177906	68180089313	68180089271	52544027621	50419040501	50102023113
	54868473100	50102022821	52544026884	52544029531	65862093488	16714034801	50419040370
	50090379300	378729853	53002169201	50090259400	54868394800	54569534900	50102010010

© 2025 Bellerose M et al. *JAMA Network Open*.

	54868474500	555901658	51862010206	430053095	53002168303	16714040601	68462031884
	52544022891	70700011787	57297084813	52544055231	16714040804	93614891	54569549302
	65862093988	68180084611	70700012184	50419040700	65862093921	16714041602	68180083871
	54868481400	65862093487	71205052628	52544084731	65862093954	378728385	54569579800
	68462073129	378730085	603752101	65862092658	52544016541	57297087513	378730685
	93542328	430053795	75854000028	50102012003	52028306	54569487801	54569582600
	54868491100	53002173506	51862002801	16714040402	93313482	42291056528	70700012085
	70518213700	781406015	50458019412	51862031803	65862094088	63187089028	54569614400
	69238158306	378729885	54868477600	378730753	51862087202	247223028	53002161601
	51862086801	68462050329	378729656	65862084992	16714036704	247217028	50419043306
	53002071906	57297088611	50102023313	16714040702	50102013000	247216928	55289088704
	50458025120	57297084311	50102015401	54868604400	555905167	555904958	430053714
	51862047006	51862056401	53002171101	51285012698	50458019100	50419040903	65862088792
	50419040375	378728253	53002173403	53002156301	50102012000	16714036304	70700010485
	68462041929	16714040501	16714007301	50458019128	55289024708	555901479	50090249400
	68180083811	59746076342	8253605	68180087773	53002162703	16714035902	430057045
	50102023311	57297087611	68462031684	68462072084	16714034603	50458017820	70518121400
	70518201000	52544027431	51862004591	54868627300	68180089411	378730653	54868473000
	68180089373	62190715	75834011584	52544029231	16714036501	43386062030	51862086803
	52544029021	57297090211	68180085473	51862086602	50458025128	378728653	50458017806
	62176115	52544028754	51862064503	76413015028	51285094388	52544005841	68180087371
	65862089828	54868502800	53002163101	76413016628	68180086613	63187045828	16714007303
	50419040973	52544006431	16714041301	52544021028	50090242900	781410352	51285012058
	555900980	51285009158	555905158	54868377200	62190700	93542358	68180088211
	555901079	68462067295	54569499800	63187005428	51862087006	65862077888	51862027906
	51285043165	16714036301	68180085711	16714034802	52544008728	17478026128	51862087003
	21695076928	52544038331	68180088011	51862048965	51862086806	555902757	51285012797
	430058114	50090251100	93330516	54569645900	93542362	51285054628	68180086671
	63629875101	42291056584	430053995	62141116	65862086494	68462031629	781558307
	378729953	430000531	75834012990	68180089471	65862077785	54868535600	35356041128
	93214062	53002162803	93532862	50419040303	54569645800	378731553	51285042410
	50102010048	68462039484	65862084988	555902542	53002173201	65862093458	54868474400
	68180085771	53002130401	781407515	50419048203	16714035901	54868594200	76388028301
	50090147800	68462067291	603760715	52544023528	65162031684	65862077686	781410315
	555902079	378729385	247226028	53002163203	57297085711	247069128	42254024228
	70700012284	51862089206	781406215	16714007304	75854060101	55045349701	50458017800
	54868477800	68462073329	68462073184	555903479	430058545	247069028	50090373300
	50090430300	50419040900	378728053	555904379	50419040970	378729285	51285012570
	42291055328	378727453	603754049	65862088688	75834011684	378727785	53002168703
	65862086694	57297084811	68180087513	16714044101	54569516100	68462030384	555902858

© 2025 Bellerose M et al. *JAMA Network Open*.

	50090215000	24090096184	51285076993	69238103106	54569627200	52544094928	54569652500
	50102013090	65862094188	16714034803	16714040604	70700010185	54868528600	254203380
	54868627200	53002169203	54868503100	50458019728	34908062056	68462065729	68180088013
	54868616200	53002169206	16714041302	68462085284	70700011884	16714041304	50102010000
	68180085411	16714036002	70700010486	50419048271	50419048201	555904758	16714040803
	51285012870	54868610000	68462030329	430058014	70700011384	54569662000	51862047101
	68462065784	247226828	54569628100	17478026028	54569579700	93807316	34908062051
	603760801	51862088601	555905058	68180084311	68180089473	68462038884	42291059028
	54868627500	247200828	51862089203	68180085413	52544021928	603762501	247069228
	378729785	53002156401	71205017028	55045378206	16714044004	52544029831	603752199
	53002162801	16714040703	54569426900	75834011629	70700011885	53002119103	247201028
	378728685	54569657300	52544055428	603766301	68180083713	781558336	54569643400
	16714040801	53002159106	378729253	57297083713	50090015901	65862093574	21695040701
	54868044300	53002172206	54569068900	54868592200	70518005900	378728785	50458019601
	603754017	52544047536	16714044102	68180084011	16714040504	555913179	69238155406
	781406615	50090323900	50102023511	76388028306	65862094187	51862001201	51862027901
	430042014	68180090413	555900942	68180089811	51862088603	50090139200	68180089311
	55887028628	68462038829	16714044104	65862077628	70700012487	75834011529	76413011828
	54868582600	70700010484	50419048272	68180091171	50458017815	50090322900	53002153901
	50102022401	51862086802	603764217	65862077786	65862092592	93330416	51862087001
	60219103107	53217012728	51285005866	50458017606	70518047400	68258500502	603760817
	63187075428	54868459000	52544026531	254203091	65862084928	24090080184	52544020431
	62179615	51862064506	50458017600	68180086413	254203373	50102012803	54868460700
	51660012786	65862092697	51862087002	65862086594	75854060202	59762159905	53002156703
	76413012128	68180086673	50458017628	68180087911	65862077887	53002177501	54569481700
	93603182	54569384400	53002154903	53002168701	65862092585	23603003	603760602
	603752517	59762159901	378730153	8111730	378730885	68180088611	378730185
	52544026829	430001005	51285009282	254203391	603752501	51862089403	57297088613
	50090342600	68180088673	53002164703	603751217	378727485	51285094288	45802084054
	54569656500	54569535800	378728756	50090247800	59651002987	16714034002	65862088628
	378728853	16714044003	603751249	60219103106	68180089713	50102012010	555902742
	68180089913	55045348506	68788633902	65862084888	68462013279	430042060	68180084811
	247201004	68180087713	51862086603	51862023801	52544096728	53002156701	53002130406
	53002177901	53002163703	66116047028	71205019128	53002161606	54868593500	54569657400
	50090488100	75854060228	430053550	16714034604	31722094528	53002162701	76413011628
	68180084071	53002163206	59651002988	68462065684	51285008787	52544014331	8253601
	65862077792	68788703602	35356036128	68180087611	31722094532	68462065629	52026106
	59651003088	23490769901	51862089202	68462065790	642747106	50419040775	53002156303
	50419040703	53002173503	54868050200	68180083873	42291055384	50419040203	16714036702
	50102023513	34908062053	68180084073	68180088671	62178100	16714034704	16714040401

© 2025 Bellerose M et al. *JAMA Network Open*.

	50102024001	53002164706	52544096691	378731585	57297087713	23586230	51862010006
	93330328	16714034804	51285008782	555900957	62141111	16714035904	54569614500
	65862089728	93330316	378728753	52544024928	42291059084	68462037684	16714036701
	65862077728	16714037003	50458019115	57297084411	68180083813	93614882	50090139500
	555900879	70700011385	52544084828	68180084613	65862089892	51862031801	378729753
	50102020013	70700012285	50102013048	16714036601	53002161603	51862028401	70700011484
	51862007201	16714040404	16714035903	63629266601	378655056	53002171106	68462030984
	603766317	51862089606	68788632502	430053914	54868486000	21695085603	430001028
	52544014328	65862077788	51862047001	555071579	65862077687	52544029841	65162031658
	50458017812	75834012929	75834013084	31722093432	53002173501	378731685	50458019706
	781565815	53002156306	23490765301	33261012001	50102015403	378727753	603761017
	68462050384	52544093628	59651003085	68180088213	50419040976	50102013003	66116043628
	65862092588	53002168306	52544023341	50419040300	555904358	53002164801	65862077886
	51862000701	65862094058	555900867	70700011585	68180083771	58016474701	603760917
	53002173206	53002172203	603763401	53002118801	54569662300	50458017615	68180086013
	23597005	52544016741	53002071901	42254026028	51862054506	57297088213	50458017828
	16714040602	65862084828	63187091128	23586228	52544063128	54868050901	68462064693
	53002173406	52544095928	70700011584	42254028728	70700011984	68462041984	93209058
	52544023328	68180083773	35356025528	50102020001	53002153906	68180085471	54569644800
	430053060	57297085413	53002160001	50090225900	50090218300	57297090213	35356036528
	54569645500	68180089171	65862092586	50090248100	55045283902	57297090311	53002119101
	51862009706	69238160706	247151728	68180084413	50102022421	57297090313	430048214
	70700011685	378729685	247176421	50090250500	68180087771	60219153106	642747101
	378728185	51862088401	54868618300	51862064502	781407552	603359001	50419040371
	50102024023	51862002806	54868485100	50419040770	68180086411	52544084728	68180090411
	53002153903	16714041601	76413010528	54868156400	52544098128	68180086611	555034458
	51285013197	53002130403	55590507	54569655600	61916009030	555904558	63187088928
	555034479	781411052	54868424000	53002177903	57297085411	68180087313	378727285
	54569643600	51862089201	51862009701	65862089928	17478026006	53002168301	68462085229
	53002145403	68180090313	16714046401	54868409300	54868231600	52544055431	68180089273
	65862093554	65862089888	62190120	54569581600	52544016528	63187074828	603760648
	51862010201	247176404	603764017	62125115	57297087511	21695077028	75834013090
	50090145600	54569487800	53002154906	16714040701	54868621000	50090260300	68462037629
	35356001468	68180086471	53002156403	51285008198	50090137600	57297087711	54569612800
	16714040802	68180091113	50102013010	53002163706	50090200600	53002071903	50090209700

© 2025 Bellerose M et al. *JAMA Network Open*.

eMethods 3. Propensity Scores

To account for differences in the characteristics of women in our sample who were eligible and ineligible for Medicaid, we calculated propensity scores with Inverse Probability of Treatment Weights.[6, 7] For our first analysis assessing the monthly probability of contraceptive use by insurance type, we calculated propensity scores using a logistic regression model with a binary outcome of monthly Medicaid enrollment and the six covariates described below, which were available and defined consistently in the TM, MA, and Medicaid claims data. We removed person-month observations missing any of these variables from our sample. We weighted women in the treatment group (enrolled in Medicaid or dual enrolled) by $\frac{proportion\ treated}{propensity\ score}$ and the control group (Medicare alone) by $\frac{proportion\ untreated}{1-propensity\ score}$.

For our second analysis evaluating the impact of gaining contraceptive coverage on contraceptive use, we used the methods described above, but with the binary outcome of being in the treatment (Medicare to dual transition) vs control group (continuous Medicare alone).

Included covariates

Age group. We included the following age groups: 20-24, 25-29, 30-34, 35-39, 40-44, and 45-49 years. No observations were missing age group.

Race and ethnicity. We used the single race/ethnicity variable included in the Medicare Current Beneficiary Summary (MBSF) file (BENE_RACE_CD) and the Medicaid Transformed Analytic Files Demographic and Eligibility file (RACE_ETHNCTY_CD). This variable is populated using an algorithm that includes language, state, and last name. It was developed to improve identification of certain groups, particularly Asian/Pacific Islanders and Hispanics, in Social Security Administration race and ethnicity data. This algorithm-based variable may not accurately classify all individuals in the study sample.[8] Roughly 22% of Medicaid person-month observations and 2% of Medicare or dual enrolled person-month observations were missing race/ethnicity and were removed from our original sample.

County-level MA penetration. We included publicly available monthly county-level MA penetration values from the Centers for Medicare and Medicaid Services (CMS): https://www.cms.gov/data-research/statistics-trends-and-reports/medicare-advantagepart-d-contract-and-enrollment-data/ma-state/county-penetration. These values are a ratio of the number people enrolled in MA in a US county over the number of people eligible for MA within that county. For observations missing a county (2.5% of the original sample), we used the monthly state average MA penetration value. No observations were missing a state.

Zip-code level median household income. We included publicly available data on median household income within a given U.S. zip code reported on the 2016 American Community Survey. For observations missing a zip code (1.5% of the sample), we used the county average median household income value. We removed observations missing a county (2.5% of the original sample).

© 2025 Bellerose M et al. *JAMA Network Open*.

Zip-code level percent living below poverty. We included publicly available data on the percent of households within a given U.S. zip code that reported incomes falling below the federal poverty line on the 2016 American Community Survey. For observations missing a zip code (1.5% of the sample), we used the county average percent living below poverty value. We removed observations missing a county (2.5% of the original sample).

Zip-code level percent with a four-year degree. We included publicly available data on the percent of the population over age 25 in a given U.S. zip code who reported having received a bachelor's degree or higher on the 2016 American Community Survey. For observations missing a zip code (1.5% of the sample), we used the county average percent with a four-year degree values. We removed observations missing a county (2.5% of the original sample).

Summary of propensity scores

Propensity scores are described in eTable 3. Propensity scores had nearly complete overlap, so we did not trim extreme weights.

eTable 3. Propensity Scores Summary

	N	Mean	P5	P25	P50	P75	P95	Min	Max
Propensity scores									
Analysis 1: Contraceptive use by insurance type									
No Medicaid	4,890,813	0.83	0.62	0.76	0.85	0.91	0.97	0.23	1.00
Medicaid	46,610,490	0.91	0.76	0.88	0.94	0.97	0.99	0.23	1.00
Analysis 2: Impact of gaining coverage on contraceptive use									
Control	1,540,812	0.55	0.41	0.47	0.53	0.62	0.73	0.24	0.86
Treatment	803,123	0.62	0.44	0.52	0.62	0.70	0.79	0.23	0.87
Inverse probability of treatment weights with stabilized weights									
Analysis 1: Contraceptive use by insurance type									
No Medicaid	4,890,813	0.57	0.14	0.23	0.35	0.60	1.75	0.07	33.09
Medicaid	46,610,490	0.57	0.52	0.53	0.55	0.59	0.68	0.52	2.21
Analysis 2: Impact of gaining coverage on contraceptive use									
Control	1,540,812	0.22	0.16	0.18	0.20	0.25	0.35	0.12	0.69
Treatment	803,123	0.24	0.17	0.19	0.23	0.27	0.38	0.12	0.73

© 2025 Bellerose M et al. *JAMA Network Open*.

Tests of common support assumption

We assessed propensity score balance visually (eFigure 2) and using standardized mean differences (eTable 4; eFigure 3). We considered the model appropriately bias-reduced as all covariate differences were less than 0.1 standardized difference after weighting.

We also examined the characteristics of propensity score outliers. In analysis 1, the majority of outlier observations from women who were enrolled in Medicaid but had low propensity scores (<5th percentile) were from White women (92.9%) aged 45-49 (68.2%) who lived in zip codes with a lower percentage of people under the poverty line compared to the full Medicaid sample (10.5%) (eTable 5). The majority of outlier observations from women who were not enrolled in Medicaid with high propensity scores (>95th percentile) were from Black women (68.5%) aged 20-24 (62.6%) who lived in zip codes with a higher percentage of people under the poverty line (32.9%) compared to the full Medicaid sample.

eFigure 2. Visual Test of Common Support for Analysis 1 (Left) and Analysis 2 (Right) Propensity Score Models

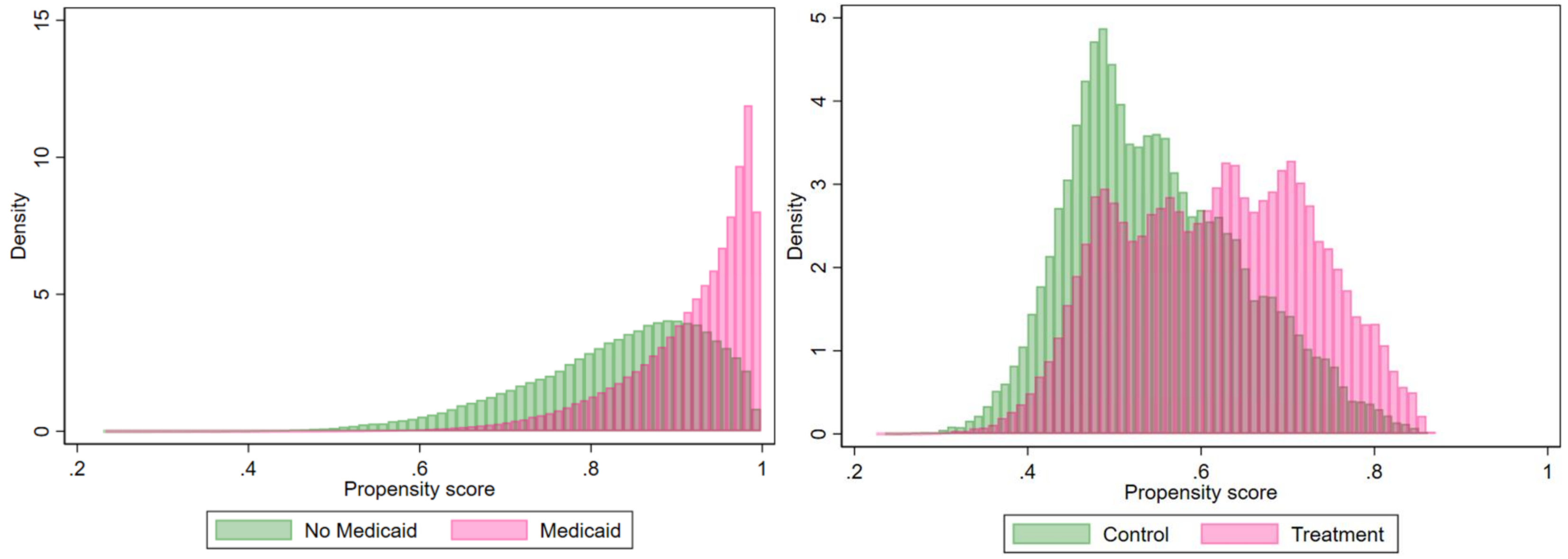

© 2025 Bellerose M et al. *JAMA Network Open*.

eTable 4. Characteristics of Groups Before and After Propensity Score Weighting

	Before weighting		After weighting		
	Control	Treatment	Control	Treatment	SMD*
Analysis 1: Contraceptive use by insurance type					
Age group %					
20-24	2.3	13.0	12.1	12.0	-0.002
25-29	4.2	15.2	14.9	14.2	-0.020
30-34	9.0	16.6	16.2	15.9	-0.007
35-39	16.4	16.9	16.8	16.8	0.001
40-44	25.9	17.2	17.6	18.0	0.010
45-49	42.2	21.1	22.5	23.1	0.014
Race/ethnicity %					
Asian	1.9	2.0	1.8	2.0	0.014
Black	17.0	30.7	30.3	29.4	-0.019
Hispanic	7.4	14.1	11.1	13.4	0.070
White	71.7	51.4	54.3	53.3	-0.020
Multiple/other races	2.0	1.8	2.4	1.8	-0.042
County-level MA penetration	35.8 (15.2)	30.1 (12.5)	30.4 (14.3)	30.6 (12.6)	0.015
Zip code level characteristics					
Median household income	55041.6 (21887.5)	48184.0 (18597.1)	48225.3 (19175.9)	48831.9 (19049.2)	0.032
% in poverty	16.5 (10.6)	20.4 (10.5)	20.2 (11.8)	20 (10.5)	-0.015
% with 4-year college degree	26.6 (14.2)	22.7 (13.1)	23 (12.6)	23.1 (13.3)	0.005
Analysis 2: Impact of gaining coverage on contraceptive use					
Age group %					
20-24	1.5	8.9	2.1	8.4	0.009
25-29	3.3	9.4	5.8	10.5	0.003
30-34	7.9	14.2	11.3	14.8	0.002
35-39	15.9	19.3	18.4	19.1	0.000
40-44	27.2	21.9	25.8	21.6	-0.003
45-49	44.3	26.3	36.5	25.6	0.000
Race/ethnicity %					0.005
Asian	1.8	1.9	1.9	1.9	0.009

© 2025 Bellerose M et al. *JAMA Network Open*.

Black	15.2	26.1	19.5	27.1	0.004
Hispanic	7.4	8.2	7.9	8.0	-0.003
White	73.6	61.4	68.4	60.5	-0.006
Multiple/other races	2.0	2.3	2.3	2.4	0.006
County-level MA penetration	36.8 (15.6)	33.8 (13.8)	36.9 (15.8)	34.8 (13.8)	-0.003
Zip code level characteristics					0.009
Median household income	55717.7 (22344.7)	51850 (19799.7)	53376.7 (21434.1)	51807 (20128.9)	0.003
% in poverty	16.2 (10.7)	18 (9.9)	17.3 (11.2)	18.1 (10.1)	0.002
% with 4-year college degree	27 (14.3)	25 (13.6)	25.9 (13.8)	25 (13.8)	0.000

* SMD = standardized mean difference

© 2025 Bellerose M et al. *JAMA Network Open*.

eFigure 3. Standardized Mean Differences for Analysis 1 Before (A) and After (B) Propensity Score Weighting and Analysis 2 Before (C) and After (D) Propensity Score Weighting

© 2025 Bellerose M et al. *JAMA Network Open*.

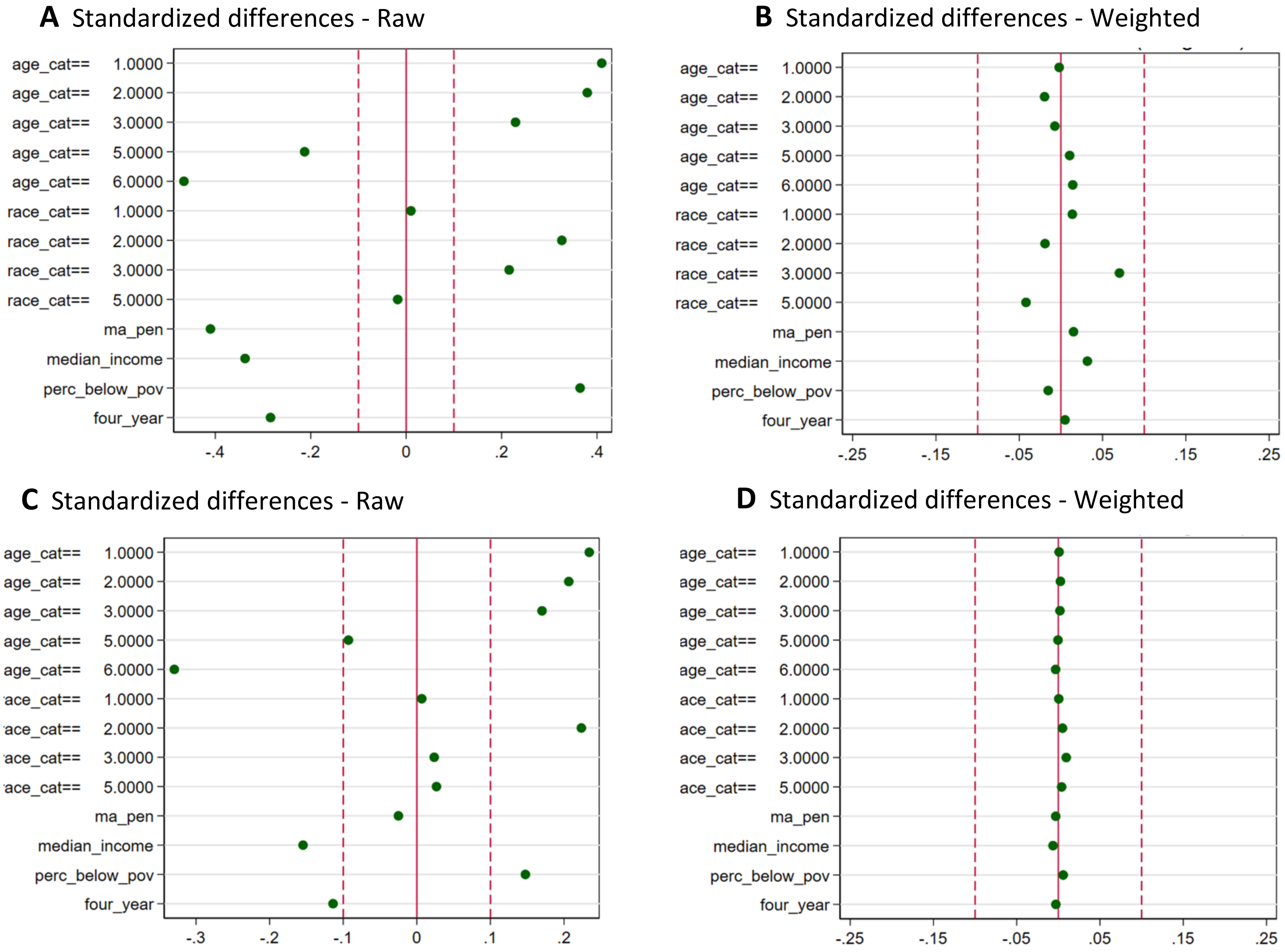

The red dotted lines in each panel represent our criteria for strong overlap of 0.1 standardized mean difference.

© 2025 Bellerose M et al. *JAMA Network Open*.

eTable 5. Characteristics of Propensity Score Outliers

	Analysis 1: contraceptive use by insurance type		Analysis 2: impact of gaining coverage on contraceptive use	
	5th percentile, treated	95th percentile, untreated	5th percentile, treated	95th percentile, untreated
Age group, %				
20-24	--	62.6	0.1	9.3
25-29	--	36.6	0.3	48.7
30-34	0.5	0.8	9.1	39.8
35-39	5.9	--	90.5	2.2
40-44	25.4	--	--	--
45-49	68.2	--	--	--
Race/ethnicity, %				
Asian	1.2	1.0	1.4	1.3
Black	2.4	68.5	0.4	75.4
Hispanic	1.1	21.2	7.3	8.1
White	92.9	6.3	90.5	8.5
Multiple/other races	2.3	3.0	0.4	6.6
County-level MA penetration	45.1 (10.6)	21.3 (9.1)	35.5 (14.0)	38.6 (16.9)
Zip code level characteristics				
Median household income	68486.0 (24149.2)	32342.1 (11318.5)	95276.4 (21701.3)	36946.0 (13259.5)
% in poverty	10.5 (5.8)	32.9 (13.2)	6.10 (3.0)	27.9 (12.8)
% with 4-year college degree	34.4 (16.5)	16.1 (8.3)	49.1 (14.2)	18.8 (9.4)

© 2025 Bellerose M et al. *JAMA Network Open*.

eFigure 4. Crude Contraceptive Method Use by Public Insurance Type

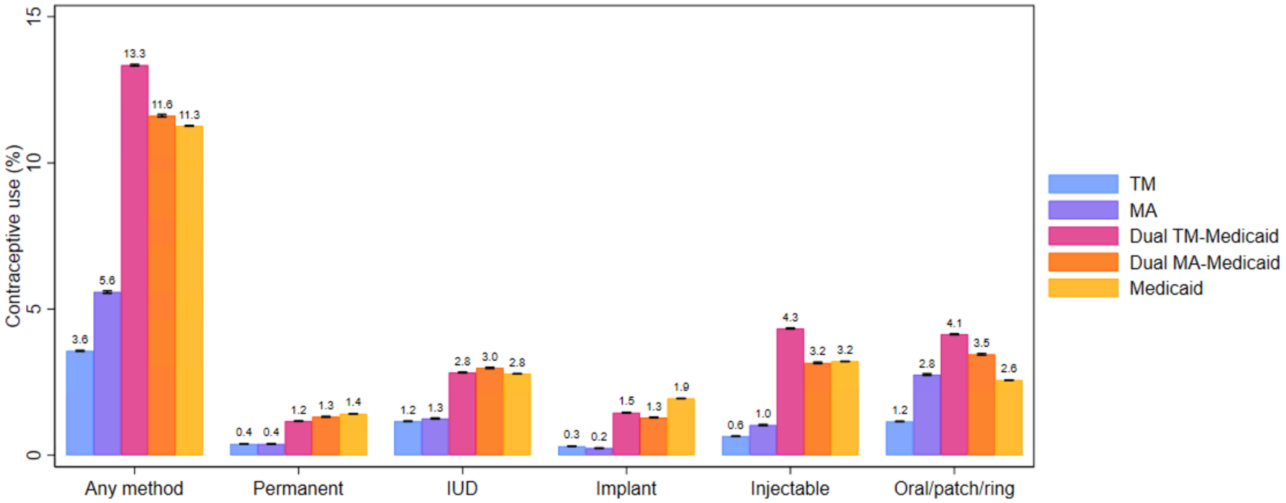

eMethods 4. Staggered Difference-in-Differences Model

Tests of model assumptions

Below, we list the sDID model assumptions and describe how we assessed each.

<u>Staggered treatment adoption</u>: <u>once a unit becomes treated, it remains treated in remaining periods.</u> We ensured that our data met this assumption by removing person-month observations from the treatment sample when a woman was: 1) not enrolled in Medicare alone before the transition period or 2) not dual enrolled after the transition period.

<u>No anticipation: individuals do not change their behavior in anticipation of the transition event</u>. When a person with Medicare under age 65 experiences a transition to dual enrollment, they should not lose coverage of any health services or need to change healthcare providers. Therefore, it is unlikely that a woman in our sample would change her contraceptive use in anticipation of this change in her insurance coverage. However, it is possible that a transition to dual enrollment could occur as a direct result of a life event that could simultaneously cause a woman to change her contraceptive use behaviors, such as marriage, divorce, job loss, or becoming a parent. While we cannot account for this possibility fully using claims data, the results of our placebo tests (described below) increased our confidence that that this was not a strong source of bias.

<u>No systematic selection into the treatment group based on anticipated outcomes: individuals do not choose to become dual enrolled in order to gain contraceptive coverage</u>. When someone with Medicare becomes dual enrolled, Medicaid immediately begins covering their monthly health insurance premiums and most of their cost-sharing requirements. Therefore, it is likely that a

© 2025 Bellerose M et al. *JAMA Network Open*.

person with Medicare would dual enroll in Medicaid ss soon as they became aware that they were eligible, rather than waiting for a healthcare need to arise.

Parallel pre-trends: changes over time in the treatment cohort would follow the same pattern as changes over time in the control cohort in the absence of treatment. We assessed parallel trends visually by examining crude use of each contraceptive method before and after a real or proxy transition among our treatment and control cohorts. Trends appeared to be approximately parallel prior to the transition for all methods with the possible exception of oral/patch/ring (eFigure 5). We also performed a logistic regression to see if there was a significant interaction between contraceptive method use and month comparing the treatment and control groups. Before and after propensity score weighting, only the oral/patch/ring interaction term was significant based on a p-value of 0.05 (eTable 6). For that method, the direction of the effect was opposite before versus after the transition (declining use in the treatment group relative to the control group before the transition and increasing use after), meaning that this source of bias would likely have attenuated the sDID results. Based on these results, we continued the analysis under the parallel trends assumption.

© 2025 Bellerose M et al. *JAMA Network Open*.

eFigure 5. Crude Contraceptive Use Before and After a Real or Proxy Transition in Insurance Coverage

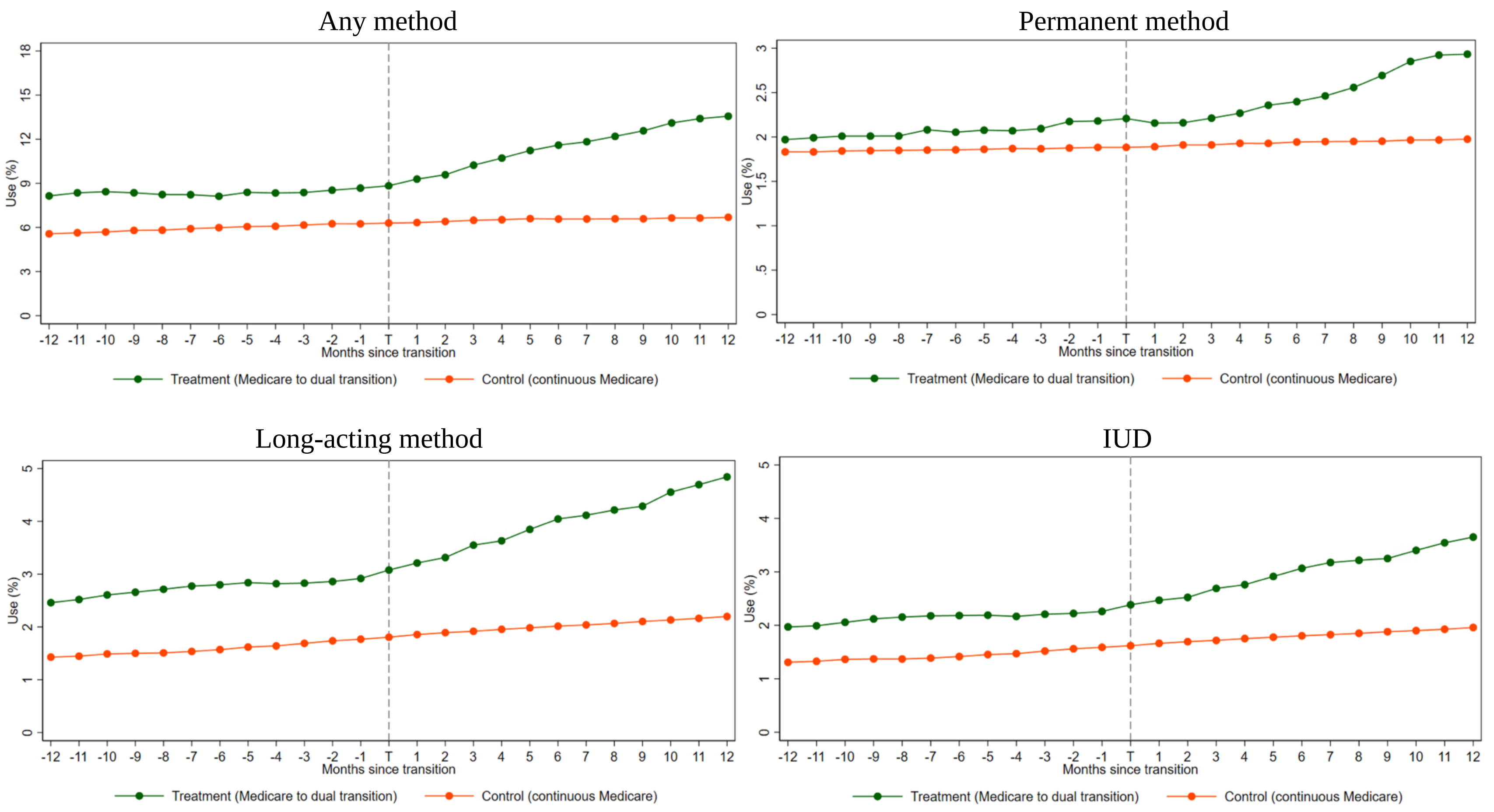

© 2025 Bellerose M et al. *JAMA Network Open*.

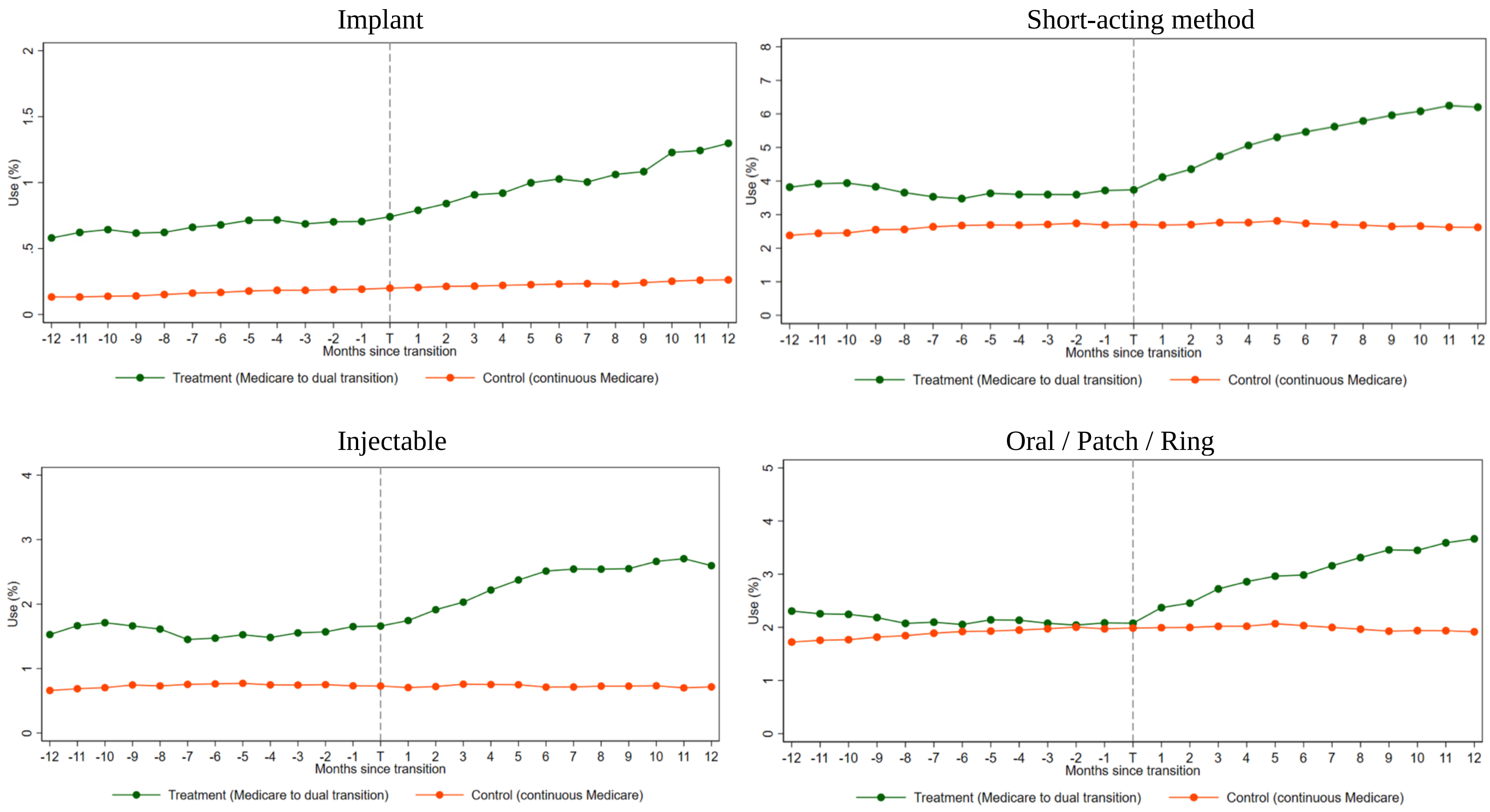

© 2025 Bellerose M et al. *JAMA Network Open*.

eTable 6. Logistic Regression of Association Between Contraceptive Method Use and Month in the Prestudy Period, Comparing the Treatment and Control Groups

(logit outcome c.month##treatment)

	Interaction term p value	
Method	No adjustment	Propensity score weighted
Any method	0.051	0.087
Permanent method	0.442	0.306
Long-acting method	0.456	0.290
IUD	0.442	0.225
Implant	0.206	0.212
Short-acting method	0.001	0.009
Injectable	0.206	0.329
Oral / patch / ring	0.003	0.002

© 2025 Bellerose M et al. *JAMA Network Open*.

Subgroup Analyses

eTable 7. Disability Type Codes

Disability category	Disability description	CCW codes	ICD-10 codes
Intellectual or developmental	Autism	Autism	F840<br>F841
	Asperger's Syndrome		F845
	Other pervasive developmental disorders		F843<br>F844<br>F848<br>F849
	Mild, moderate, and severe intellectual disabilities	Intellectual or developmental disability<br><br>Learning disability<br><br>Other developmental disability	F700<br>F701<br>F708<br>F709<br>F710<br>F711<br>F718<br>F719<br>F720<br>F721<br>F728<br>F729<br>F730<br>F731<br>F738<br>F739<br>F780<br>F781<br>F788<br>F789<br>F790<br>F791<br>F798<br>F799
	Cerebral palsy	Cerebral palsy	G80
	Genetic disorder	Cystic fibrosis	E791<br>F842<br>Q851<br>Q871<br>Q8723<br>Q8731

© 2025 Bellerose M et al. *JAMA Network Open*.

			Q900<br>Q901<br>Q902<br>Q909<br>Q910<br>Q911<br>Q912<br>Q913<br>Q914<br>Q915<br>Q916<br>Q917<br>Q934<br>Q9351<br>Q9359<br>Q937<br>Q938<br>Q939<br>Q960<br>Q964<br>Q968<br>Q992<br>Q998Q999
	Alzheimer’s disease or related dementias		G300<br>G301<br>G308<br>G309<br>G3101<br>G3109<br>G311<br>G312<br>G318<br>G320<br>G3281<br>G3289<br>F01<br>F02<br>F03
	Other	Brain injury	F843<br>F844<br>P043<br>Q860<br>Q861<br>Q878
Physical	Cerebral palsy	Cerebral palsy	G800<br>G801

© 2025 Bellerose M et al. *JAMA Network Open*.

			G802<br>G803<br>G804<br>G808<br>G809<br>G8381
	Spina bifida	Spina bifida	Q050<br>Q051<br>Q052<br>Q054<br>Q055<br>Q056<br>Q057<br>Q059
	Spinal cord injury	Spinal cord injury	S14<br>S24<br>S34<br>G834<br>G835<br>G959
	Muscular dystrophy, multiple sclerosis, other mobility impairments or chronic pain	Muscular dystrophy<br>Multiple sclerosis<br>Mobility impairment<br>Fibromyalgia	G121<br>G129<br>G35<br>G7000<br>G7101<br>G7102<br>G7109<br>G7111<br>G7121<br>G7122<br>G7129<br>M0800<br>M0809<br>M3320<br>M3329
	Hemiplegia, Paraplegia, quadriplegia		G810<br>G811<br>G819<br>G8220<br>G8221<br>G8222<br>G825<br>G830<br>G831<br>G823<br>G834

© 2025 Bellerose M et al. *JAMA Network Open*.

			G839<br>I680<br>I691<br>I692<br>I693<br>I698<br>I699<br>M623
	Epilepsy	Epilepsy	G400<br>G401<br>G402<br>G403<br>G405<br>G408<br>G409<br>G40A<br>G4089
	Parkinson’s	Parkinson’s	G20A1<br>G20A2<br>G20B1<br>G20B2<br>G20C<br>G2111<br>G2119<br>G212<br>G213<br>G214<br>G219
	Osteoporosis	Osteoporosis	
	Other		A5206<br>A8100<br>A8101<br>A8109<br>A811<br>A812<br>A8181<br>A8182<br>A819<br>B150<br>B160<br>B162<br>B170<br>B1711<br>B172<br>B178
Sensory	Vision impairment	Visual impairment	H4710

© 2025 Bellerose M et al. *JAMA Network Open*.

			H47619<br>H47639<br>H540<br>H541<br>H542<br>H543<br>H544<br>H545<br>H546<br>H547<br>H548<br>Q111<br>A158
	Speech disorder		F940<br>R481
	Deaf or hard of hearing	Hearing impairment	H90<br>H91<br>Q16
Mental health related	Anxiety	Anxiety	F4
	Depression	Depression	
	Schizophrenia	Schizophrenia	F2
	Personality disorders	Bipolar disorder	F6
	Mental health disorder due to substance use		F1
	Physiological disturbances		F5
	Mood disorder		F3
	PSTD	PSTD	
	Self harm		E950<br>E951<br>E952
	Other		F9

© 2025 Bellerose M et al. *JAMA Network Open*.

Contraceptive use by disability type

eFigure 6. Propensity Score–Weighted Contraceptive Use by Public Insurance and Disability Type

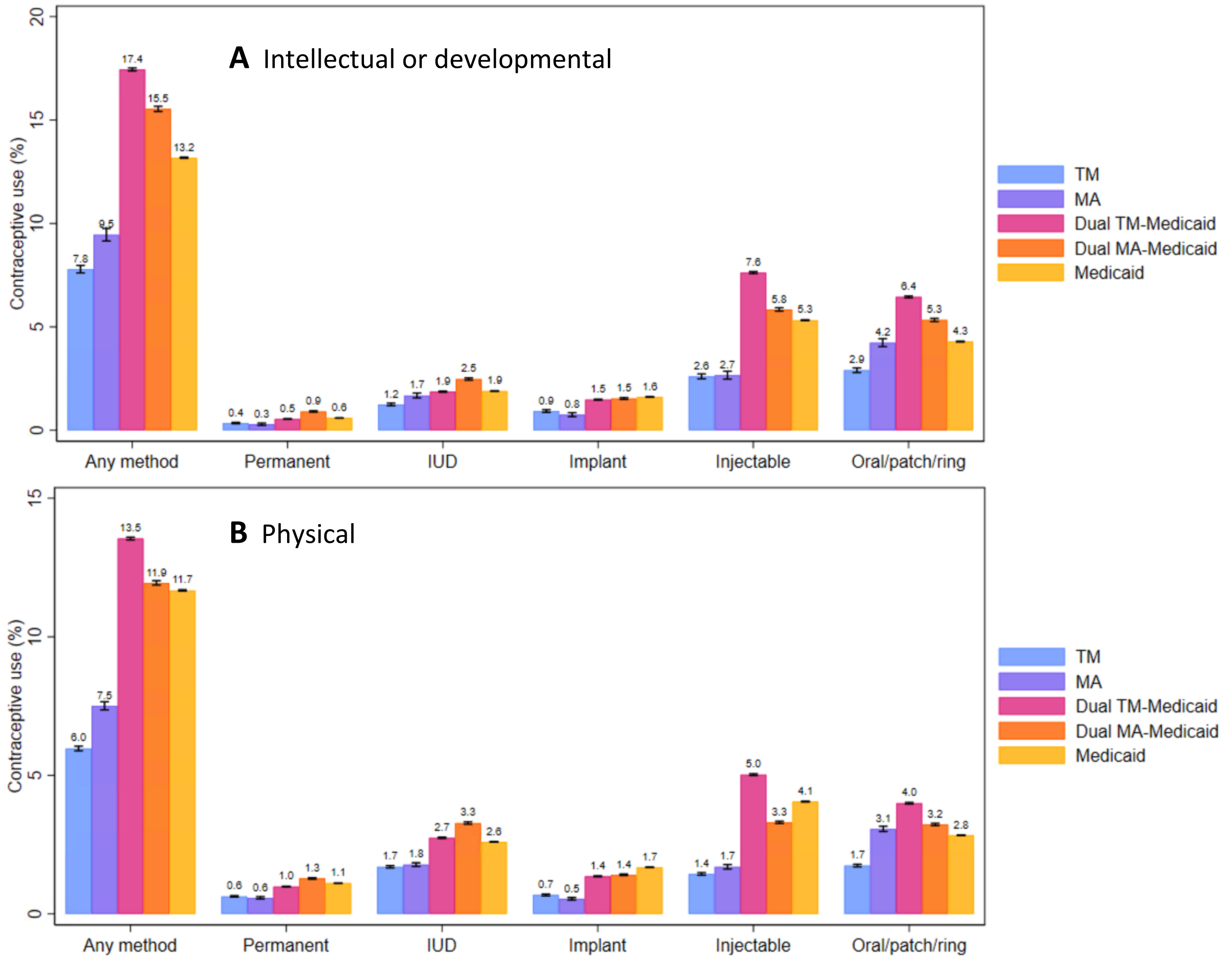

© 2025 Bellerose M et al. *JAMA Network Open*.

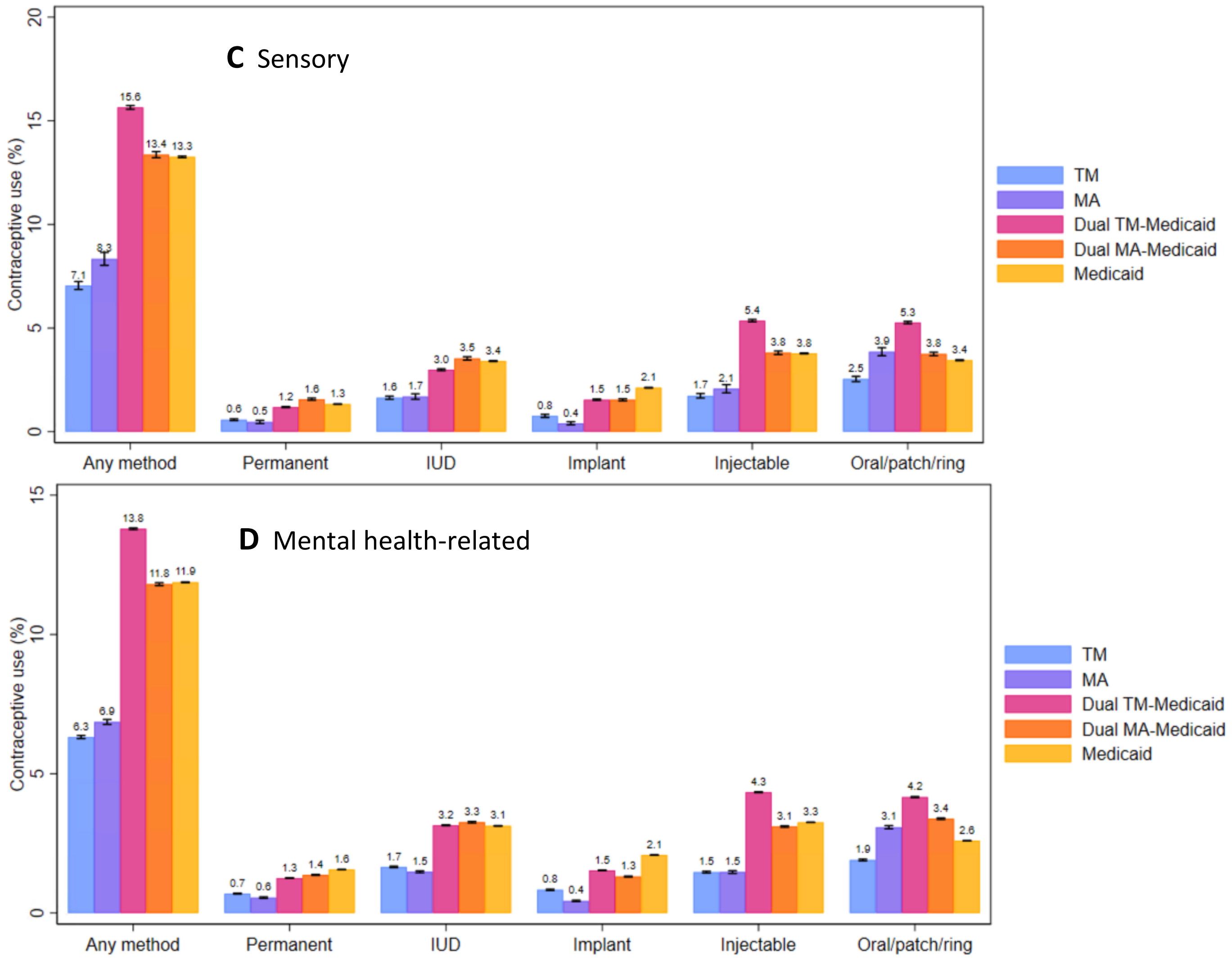

© 2025 Bellerose M et al. *JAMA Network Open*.

Impact of gaining coverage on contraceptive use by disability type

eTable 8. sDID Results by Disability Type

	Pre-transition use, % (95% CI)		Post-transition use, % (95% CI)		Percentage point change (95% CI)	
	12-month average	Month before transition	12-month average	12 months after transition	12-month average	12 months after transition
IDD						
Any method	11.3 (10.9, 11.6)	12.7 (11.8, 13.6)	16.3 (16.0, 16.6)	18.5 (17.3, 19.6)	4.5 (3.6, 5.3)	6.1 (4.7, 7.5)
Permanent method	0.9 (0.8, 1.0)	1.0 (0.7, 1.2)	1.2 (1.1, 1.3)	1.4 (1.0, 1.7)	-0.0 (-0.2, 0.1)	--
Long-acting method	2.5 (2.3, 2.6)	2.7 (2.2, 3.1)	3.3 (3.2, 3.5)	4.1 (3.5, 4.7)	0.7 (0.3, 1.0)	--
IUD	1.5 (1.4, 1.6)	1.5 (1.2, 1.9)	2.1 (2.0, 2.2)	2.5 (2.1, 3.0)	0.3 (0.0, 0.5)	--
Implant	1.1 (1.0, 1.2)	1.3 (1.0, 1.6)	1.4 (1.3, 1.5)	1.7 (1.3, 2.1)	0.4 (0.2, 0.6)	--
Short-acting method	8.1 (7.8, 8.4)	9.3 (8.5, 10.1)	12.0 (11.8, 12.3)	13.5 (12.5, 14.5)	4.0 (3.2, 4.9)	5.1 (3.8, 6.4)
Injectable	3.9 (3.7, 4.1)	4.7 (4.1, 5.3)	6.0 (5.8, 6.1)	6.9 (6.2, 7.7)	1.8 (1.3, 2.3)	2.5 (1.7, 3.4)
Oral / patch / ring	4.3 (4.1, 4.5)	4.6 (4.1, 5.2)	6.1 (6.0, 6.3)	6.6 (5.9, 7.3)	2.3 (1.6, 2.9)	2.6 (1.6, 3.6)
Physical disability						
Any method	8.8 (8.6, 9.0)	9.8 (9.3, 10.3)	11.9 (11.7, 12.0)	13.8 (13.1, 14.5)	2.9 (2.5, 3.3)	4.0 (3.3, 4.7)
Permanent method	2.1 (2.0, 2.2)	2.1 (1.9, 2.3)	2.3 (2.3, 2.4)	2.5 (2.2, 2.9)	0.1 (0.0, 0.1)	--
Long-acting method	2.7 (2.6, 2.8)	3.2 (2.9, 3.5)	3.8 (3.8, 3.9)	4.8 (4.4, 5.3)	0.8 (0.6, 1.0)	1.4 (1.0, 1.7)
IUD	2.0 (1.9, 2.1)	2.4 (2.1, 2.6)	3.0 (2.9, 3.1)	3.6 (3.2, 4.0)	0.5 (0.3, 0.7)	0.8 (0.5, 1.2)
Implant	0.8 (0.7, 0.8)	0.9 (0.8, 1.1)	1.0 (0.9, 1.0)	1.3 (1.1, 1.5)	0.3 (0.2, 0.4)	0.6 (0.4, 0.8)
Short-acting method	4.3 (4.2, 4.5)	4.8 (4.4, 5.2)	6.1 (6.0, 6.2)	7.0 (6.5, 7.5)	2.2 (1.8, 2.5)	2.8 (2.2, 3.5)
Injectable	2.1 (2.0, 2.2)	2.5 (2.2, 2.8)	2.9 (2.8, 3.0)	3.6 (3.2, 4.0)	0.8 (0.6, 1.1)	1.3 (0.9, 1.7)
Oral / patch / ring	2.3 (2.2, 2.4)	2.4 (2.1, 2.6)	3.2 (3.1, 3.3)	3.4 (3.1, 3.8)	1.4 (1.1, 1.7)	1.6 (1.1, 2.1)
Sensory						
Any method	10.9 (10.4, 11.3)	12.4 (11.2, 13.6)	13.5 (13.2, 13.9)	15.7 (14.2, 17.2)	2.2 (1.3, 3.1)	3.8 (2.2, 5.4)
Permanent method	2.1 (1.9, 2.2)	2.3 (1.8, 2.8)	2.5 (2.3, 2.6)	2.5 (1.8, 3.2)	0.1 (0.0, 0.2)	--
Long-acting method	3.3 (3.0, 3.5)	3.7 (3.0, 4.5)	4.0 (3.8, 4.2)	4.7 (3.8, 5.6)	0.5 (0.1, 1.0)	--
IUD	2.3 (2.1, 2.5)	2.6 (2.0, 3.2)	3.0 (2.8, 3.2)	3.5 (2.8, 4.3)	0.4 (0.0, 0.9)	--
Implant	1.1 (0.9, 1.2)	1.3 (0.9, 1.8)	1.2 (1.1, 1.3)	1.4 (0.9, 1.8)	0.1 (-0.1, 0.4)	--
Short-acting method	5.9 (5.6, 6.2)	7.0 (6.1, 8.0)	7.5 (7.2, 7.8)	8.9 (7.7, 10.1)	1.5 (0.7, 2.4)	2.8 (1.3, 4.2)

© 2025 Bellerose M et al. *JAMA Network Open*.

Injectable	2.7 (2.4, 2.9)	3.3 (2.6, 4.0)	3.6 (3.4, 3.8)	4.2 (3.4, 5.0)	0.6 (0.1, 1.2)	0.6 (-0.3, 1.6)
Oral / patch / ring	3.3 (3.0, 3.5)	3.9 (3.2, 4.6)	4.0 (3.8, 4.2)	4.8 (3.9, 5.7)	0.8 (0.2, 1.5)	2.1 (0.9, 3.2)
Mental health related						
Any method	9.6 (9.5, 9.8)	11.1 (10.8, 11.5)	12.8 (12.7, 12.9)	14.9 (14.4, 15.4)	2.6 (2.4, 2.9)	3.8 (3.3, 4.2)
Permanent method	2.1 (2.0, 2.2)	2.4 (2.2, 2.5)	2.7 (2.6, 2.7)	2.9 (2.7, 3.2)	0.1 (0.0, 0.1)	0.1 (0.1, 0.2)
Long-acting method	3.3 (3.2, 3.3)	3.9 (3.7, 4.1)	4.4 (4.4, 4.5)	5.4 (5.1, 5.7)	0.8 (0.7, 0.9)	1.4 (1.1, 1.6)
IUD	2.3 (2.2, 2.4)	2.7 (2.5, 2.9)	3.3 (3.2, 3.3)	3.9 (3.6, 4.1)	0.5 (0.4, 0.6)	0.8 (0.6, 1.0)
Implant	1.1 (1.0, 1.1)	1.3 (1.2, 1.5)	1.3 (1.3, 1.4)	1.7 (1.5, 1.8)	0.4 (0.3, 0.4)	0.6 (0.5, 0.8)
Short-acting method	4.6 (4.5, 4.6)	5.2 (5.0, 5.5)	6.2 (6.1, 6.2)	7.1 (6.8, 7.5)	1.9 (1.6, 2.1)	2.5 (2.1, 2.9)
Injectable	2.3 (2.2, 2.3)	2.7 (2.5, 2.9)	2.8 (2.8, 2.9)	3.4 (3.2, 3.6)	0.7 (0.5, 0.8)	1.0 (0.7, 1.2)
Oral / patch / ring	2.3 (2.3, 2.4)	2.6 (2.4, 2.8)	3.4 (3.3, 3.4)	3.8 (3.5, 4.0)	1.2 (1.0, 1.4)	1.6 (1.2, 1.9)

Missing cells indicate that there were not enough observations to produce a reliable effect estimate.

© 2025 Bellerose M et al. *JAMA Network Open*.

eMethods 5. Sensitivity Analyses

<u>Changes to study sample and data</u>

1. Remove observations from March-December 2020

The COVID-19 Public Health Emergency, which began in March 2020 and continued through the end of our study period, may have changed contraceptive use by influencing pregnancy desires or access to healthcare. We performed a sensitivity analysis removing observations from March through December 2020. The predicted probabilities of contraceptive use by insurance type remained similar (eFigure 7) and the impact of gaining coverage was slightly larger in magnitude at 4.3 percentage points increase (95% CI 3.8, 4.7) within 12 months (eTable 9).

eFigure 7. Estimated Probability of Contraceptive Use, January 2016 to February 2020

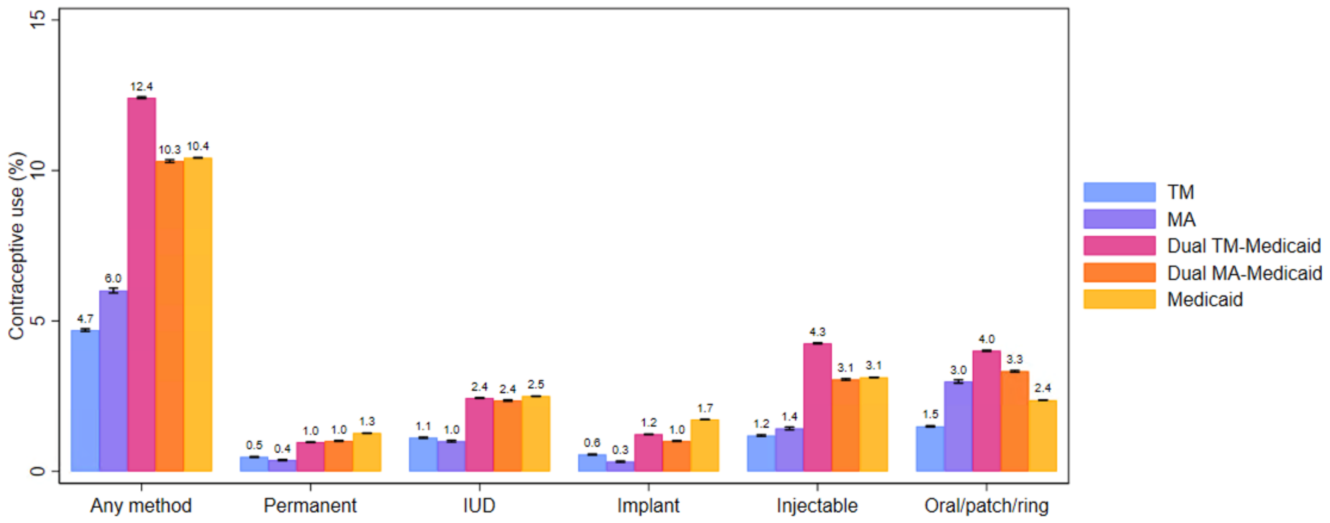

eTable 9. sDID results, January 2016 to February 2020

	Pre-transition use, % (95% CI)		Post-transition use, % (95% CI)		Percentage point change (95% CI)	
	12-month average	Month before transition	12-month average	12 months after transition	12-month average	12 months after transition
Any method	8.6 (8.4, 8.7)	9.9 (9.6, 10.2)	11.7 (11.6, 11.8)	13.8 (13.3, 14.3)	2.9 (2.6, 3.1)	4.3 (3.8, 4.7)
Permanent method	1.8 (1.8, 1.9)	2.1 (1.9, 2.2)	2.3 (2.2, 2.3)	2.5 (2.3, 2.7)	0.1 (0.0, 0.1)	0.1 (0.1, 0.2)
Long-acting method	2.7 (2.7, 2.8)	3.3 (3.1, 3.5)	3.8 (3.7, 3.8)	4.7 (4.4, 5.0)	0.8 (0.7, 0.9)	1.4 (1.2, 1.6)
IUD	2.0 (1.9, 2.0)	2.3 (2.2, 2.5)	2.7 (2.7, 2.8)	3.4 (3.1, 3.6)	0.5 (0.4, 0.6)	0.8 (0.6, 1.0)
Implant	0.9 (0.8, 0.9)	1.1 (1.0, 1.2)	1.1 (1.1, 1.2)	1.5 (1.3, 1.6)	0.4 (0.3, 0.4)	0.7 (0.5, 0.8)
Short-acting method	4.2 (4.1, 4.3)	4.8 (4.6, 5.1)	6.0 (5.9, 6.0)	7.1 (6.7, 7.4)	2.1 (1.8, 2.3)	2.9 (2.5, 3.3)
Injectable	2.1 (2.0, 2.1)	2.5 (2.3, 2.7)	2.8 (2.8, 2.9)	3.5 (3.2, 3.7)	0.8 (0.7, 1.0)	1.3 (1.0, 1.6)
Oral / patch / ring	2.2 (2.1, 2.2)	2.3 (2.2, 2.5)	3.2 (3.1, 3.2)	3.7 (3.4, 3.9)	1.3 (1.1, 1.4)	1.7 (1.3, 2.0)

© 2025 Bellerose M et al. *JAMA Network Open*.

2. Include look-back data

We created a six year look back period to identify recent receipt of a long-acting or permanent contraceptive method that would continue into the main analysis period using two data sources: 2010-2015 TM claims from a 20% random national sample of enrollees and 2010-2015 Medicaid claims from a 100% national sample of enrollees from 14 states (NY CA FL TX MD NJ PA IL GA NC VA LA OH MA). High quality MA data were not available prior to 2016.

First, we assessed contraceptive use within the main study sample, including any information on contraceptive method use occurring between 2010-2015 that would continue into the main study period (eFigure 8). We found that the predicted probability of contraceptive use was higher among women with all five insurance types after adding available look-back data, but primarily those enrolled in TM, MA, and dual enrolled. This trend was driven by higher identification of permanent and long-acting contraceptive methods within TM and MA claims. Adding look-back data to the sDID sub-sample had little impact on the sDID effect estimates (eTable 10).

We also tested the impact of restricting our sample to people with no 2010-2015 look-back data and 1, 3, and 6 years of look-back data. We found that restricting the sample to people with look-back data reduced the effect sizes to 2.0-2.2 percentage point increases by month 12. These results should be interpreted with caution, as we only had access to Medicaid data from 14 states, which may have a different landscape of contraceptive access and safety net care and no MA data.

eFigure 8. Estimated Probability of Contraceptive Use Within the Main Analysis Sample Between 2016 and 2020, Including Any Available 2010-2015 Look-Back Data

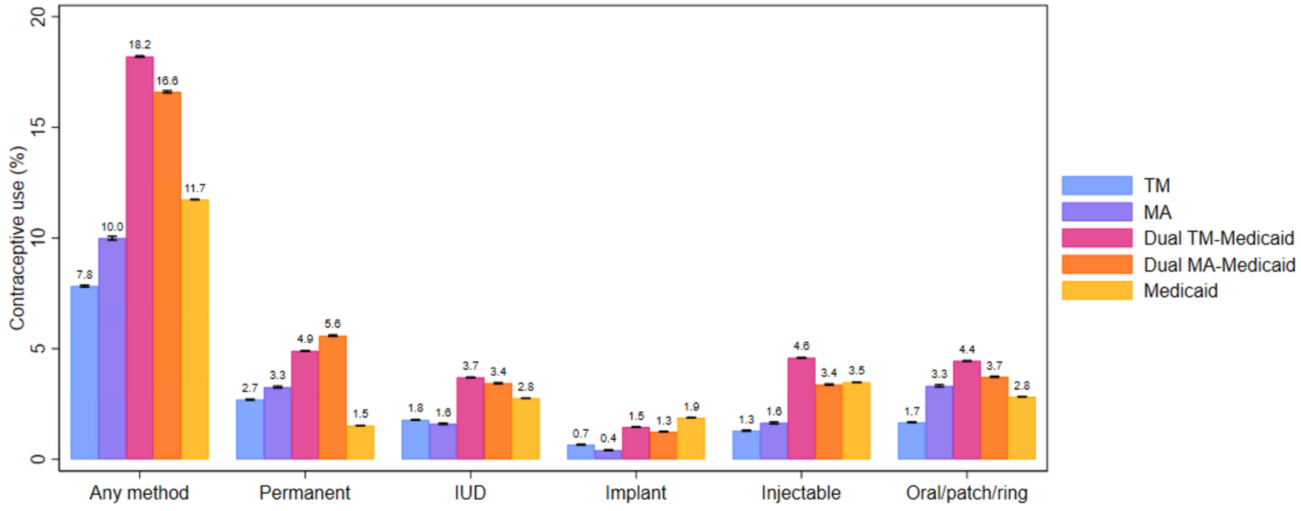

© 2025 Bellerose M et al. *JAMA Network Open*.

eTable 10. sDID Results Including Look-Back Data

Years of look-back data	N (monthly observations)		Pre transition		Post transition		Percentage point change (95% CI) –	
	Treatment	Control	12 month average	Month before transition	12 month average	12 months after transition	12 month average	12 months after transition
Any	803,123	1,540,812	8.5	9.9	11.8	14.2	2.9 (2.7, 3.1)	4.0 (3.7, 4.4)
0 (2016-2020)	94,704	349.547	6.4	7.7	10.0	12.7	3.5 (3.2, 3.8)	4.8 (4.3, 5.4)
1 (2015-2020)	183,677	1,005,395	12.3	14.2	14.4	16.1	1.3 (0.9, 1.7)	2.2 (1.5, 2.9)
3 (2013-2020)	178,233	946,836	12.7	14.5	14.6	16.3	1.3 (0.9, 1.6)	2.2 (1.5, 2.9)
6 (2010-2020)	85,836	412,133	15.4	16.9	17.3	18.6	1.4 (0.8, 1.9)	2.0 (0.9, 3.0)

© 2025 Bellerose M et al. *JAMA Network Open*.

3. Stratify results by presence of indicator for medical condition treated using contraceptives

TM and MA offer full coverage of contraceptives used to treat medical conditions on a case-by-case basis. These conditions include endometriosis, pre- and postmenopausal bleeding, polycystic ovarian syndrome, premenstrual dysphoric disorder, premenstrual tension syndrome, menorrhagia, dysmenorrhea, amenorrhea, irregular menstruation, menstrual pain, ovulation bleeding, and menstrual cramps.[9] We conducted a sensitivity analysis restricting the sample to people who had and did not have a clinical indication for one of these conditions. The codes used to identify these conditions are listed below in eTable 11. The predicted probability of any contraceptive use was higher among women with a clinical indication for contraceptive use enrolled in all five insurance types compared to the full sample (eFigure 9). In particular, the predicted probability of any contraceptive method use was 14.8% among women enrolled in TM with a clinical indication compared to 4.9% among the full sample and 3.9% among women with no clinical indication (eFigure 10). In these groups, experiencing a transition from Medicare alone to dual enrollment led to similar increase in any contraceptive use of 3.2 to 3.3 percentage points (eTable 12).

eTable 11. Codes for Clinical Indications for Contraceptives

	ICD-10 diagnosis codes
Endometriosis	N80
Pre- and post-menopausal bleeding	N924<br>N950
Polycystic ovarian syndrome (PCS)	E282
Premenstrual dysphoric disorder	F3281
Premenstrual tension syndrome	N943
Menorrhagia	N920<br>N921<br>N922
Dysmenorrhea	N944<br>N945<br>N946
Amenorrhea	N912
Irregular menstruation or pain with menstruation	N926<br>N949<br>N923<br>N940

© 2025 Bellerose M et al. *JAMA Network Open*.

eFigure 9. Estimated Probability of Contraceptive Among Women With a Clinical Indication for Contraception Use

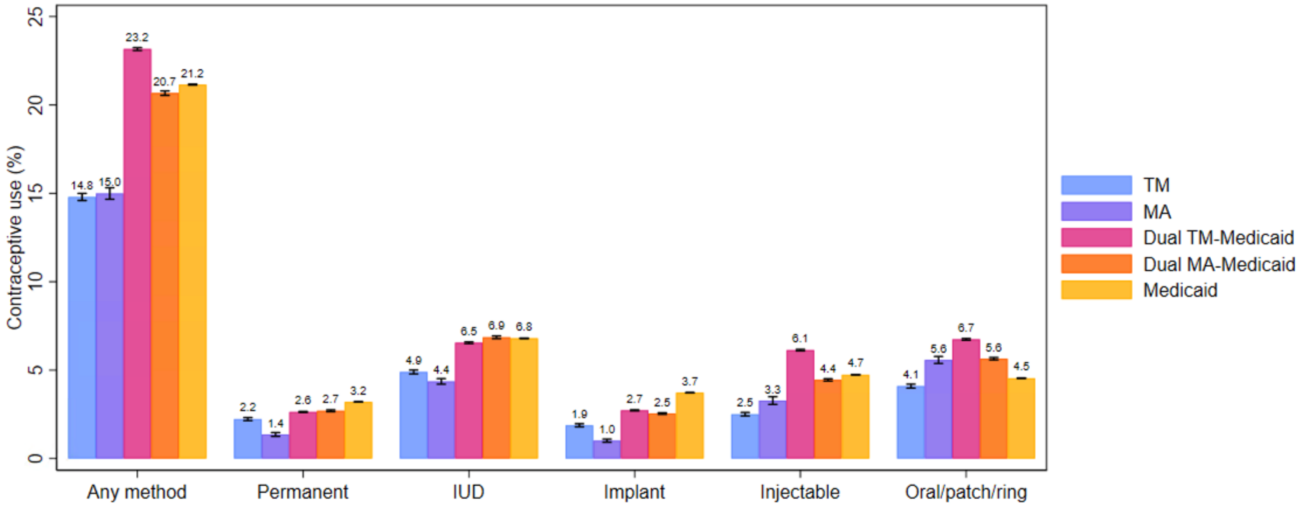

© 2025 Bellerose M et al. *JAMA Network Open*.

eFigure 10. Estimated Probability of Contraceptive Use Among Women Without a Clinical Indication for Contraception Use

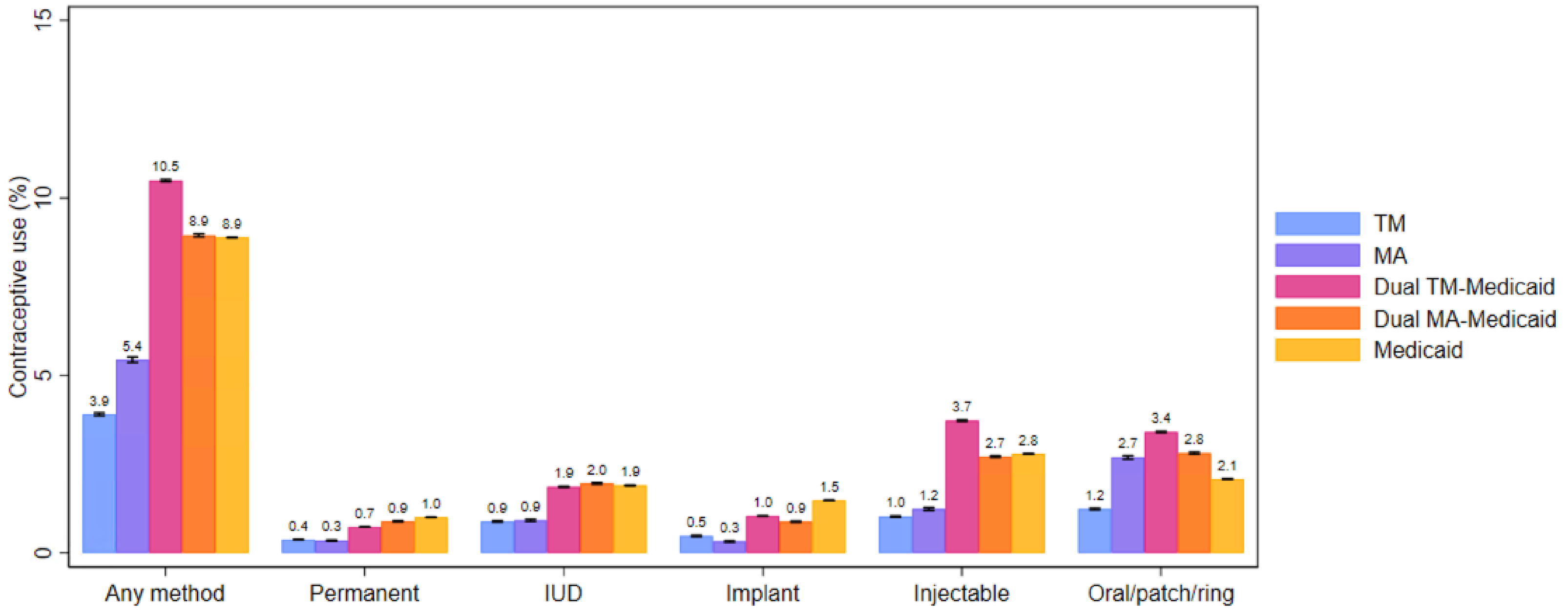

© 2025 Bellerose M et al. *JAMA Network Open*.

eTable 12. sDID Results Among Women With and Without a Clinical Indication for Contraceptive Use

	Pre-transition use, % (95% CI)		Post-transition use, % (95% CI)		Percentage point change (95% CI)	
	12-month average	Month before transition	12-month average	12 months after transition	12-month average	12 months after transition
Clinical indication (n=16,466 women with 226,486 observations)						
Any method	20.2 (19.8, 20.7)	21.4 (20.2, 22.6)	22.0 (21.7, 22.3)	23.5 (22.3, 24.6)	2.2 (1.3, 3.0)	3.2 (1.7, 4.7)
Permanent method	3.4 (3.2, 3.6)	3.6 (3.1, 4.1)	3.5 (3.4, 3.6)	3.5 (3.0, 4.0)	0.1 (-0.1, 0.2)	--
Long-acting method	8.3 (8.0, 8.7)	9.2 (8.3, 10.0)	9.1 (8.9, 9.3)	9.6 (8.8, 10.4)	0.8 (0.4, 1.2)	1.4 (0.6, 2.1)
IUD	5.9 (5.7, 6.2)	6.7 (6.0, 7.4)	7.0 (6.8, 7.1)	7.1 (6.4, 7.8)	0.4 (0.1, 0.8)	0.9 (0.2, 1.5)
Implant	2.8 (2.6, 3.0)	3.0 (2.4, 3.5)	2.5 (2.4, 2.7)	2.9 (2.5, 3.4)	0.5 (0.2, 0.7)	--
Short-acting method	9.5 (9.1, 9.8)	9.7 (8.9, 10.6)	10.5 (10.2, 10.7)	11.5 (10.7, 12.4)	1.4 (0.6, 2.2)	1.9 (0.6, 3.3)
Injectable	4.5 (4.2, 4.7)	4.8 (4.2, 5.4)	4.6 (4.5, 4.8)	5.1 (4.5, 5.7)	0.4 (-0.1, 0.9)	0.5 (-0.4, 1.3)
Oral / patch / ring	5.1 (4.9, 5.4)	5.1 (4.5, 5.7)	5.9 (5.8, 6.1)	6.6 (5.9, 7.2)	1.0 (0.3, 1.6)	1.5 (0.4, 2.6)
No clinical indication (n= 100,429 women with 1,841,579 observations)						
Any method	7.3 (7.2, 7.4)	8.4 (8.1, 8.7)	9.9 (9.8, 10.0)	11.7 (11.3, 12.2)	2.4 (2.1, 2.6)	3.3 (2.9, 3.7)
Permanent method	1.6 (1.6, 1.7)	1.8 (1.7, 1.9)	2.1 (2.1, 2.1)	2.4 (2.2, 2.6)	0.0 (0.0, 0.1)	--
Long-acting method	2.2 (2.1, 2.2)	2.6 (2.4, 2.8)	2.9 (2.9, 3.0)	3.7 (3.4, 3.9)	0.6 (0.5, 0.7)	1.1 (0.9, 1.3)
IUD	1.5 (1.5, 1.6)	1.8 (1.6, 1.9)	2.1 (2.1, 2.1)	2.6 (2.4, 2.8)	0.4 (0.3, 0.5)	0.6 (0.5, 0.8)
Implant	0.7 (0.6, 0.7)	0.9 (0.8, 1.0)	0.9 (0.9, 0.9)	1.2 (1.0, 1.3)	0.3 (0.2, 0.3)	0.5 (0.3, 0.6)
Short-acting method	3.6 (3.6, 3.7)	4.2 (4.0, 4.4)	5.1 (5.0, 5.2)	6.0 (5.6, 6.3)	1.8 (1.5, 2.0)	2.3 (1.9, 2.6)
Injectable	1.8 (1.7, 1.9)	2.2 (2.0, 2.3)	2.4 (2.3, 2.4)	2.9 (2.7, 3.1)	0.7 (0.5, 0.8)	0.9 (0.7, 1.1)
Oral / patch / ring	1.9 (1.8, 1.9)	2.1 (1.9, 2.2)	2.8 (2.7, 2.8)	3.1 (2.9, 3.3)	1.1 (0.9, 1.3)	1.4 (1.1, 1.6)

© 2025 Bellerose M et al. *JAMA Network Open*.

Changes to model specifications

4. Covariate adjustment instead of propensity score weighting

In the main paper, we present IPTW propensity score-weighted probabilities of contraceptive use. We chose this approach over standard covariate adjustment for the following reasons: 1) Since propensity scores weighting relies on the estimated probability of treatment (enrollment in Medicaid) rather than directly including each covariate in the model, using propensity scores can reduce model dependency on the functional form of each covariate and therefore be less sensitive to misspecification. 2) Since propensity score weighting summarizes information from covariates into a single score, there is less risk of multicollinearity. This could be of particular concern for our zip code level education, poverty, and household income measures, which are highly correlated. However, in some situations, standard covariate adjustment has been shown to perform better than IPTW approaches.[10] Therefore, we performed a sensitivity analysis using standard covariate adjustment. eFigure 11 shows the probabilities of contraceptive use from logistic regression models adjusted for age category, race / ethnicity, county-level MA penetration, zip code-level median household income, zip code-level percent under the federal poverty line, and zip code-level percent with a four-year college degree. eTable 13 shows sDID results with covariate adjustment. The results were very similar in magnitude to the propensity score-weighted estimates.

eFigure 11. Covariate-Adjusted Contraceptive Use

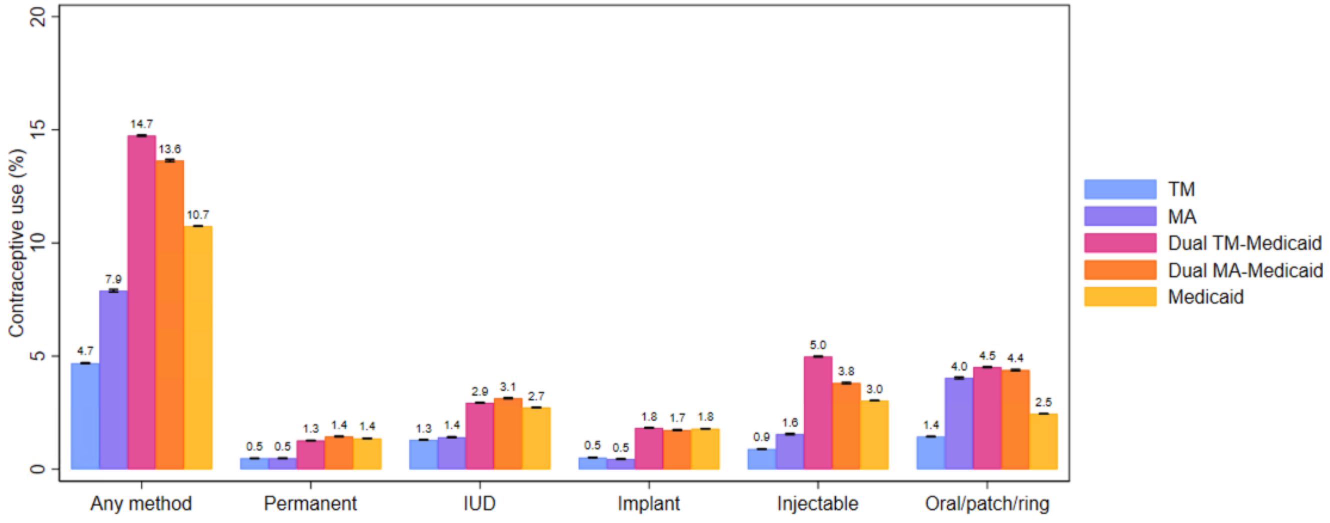

© 2025 Bellerose M et al. *JAMA Network Open*.

eTable 13. sDID Results Using Covariate Adjustment

	Pre-transition use, % (95% CI)		Post-transition use, % (95% CI)		Percentage point change (95% CI)	
	12-month average	Month before transition	12-month average	12 months after transition	12-month average	12 months after transition
Any method	7.2 (7.1, 7.2)	8.2 (7.9, 8.4)	11.2 (11.1, 11.3)	13.2 (12.8, 13.6)	2.6 (2.4, 2.9)	3.7 (3.3, 4.2)
Permanent method	1.9 (1.8, 1.9)	2.1 (1.9, 2.2)	2.3 (2.3, 2.4)	2.7 (2.5, 2.9)	0.1 (0.0, 0.1)	0.1 (0.1, 0.2)
Long-acting method	2.3 (2.2, 2.3)	2.7 (2.6, 2.8)	3.7 (3.6, 3.7)	4.6 (4.3, 4.8)	0.8 (0.6, 0.9)	1.3 (1.1, 1.6)
IUD	1.8 (1.7, 1.8)	2.1 (1.9, 2.2)	2.8 (2.7, 2.8)	3.4 (3.2, 3.6)	0.5 (0.4, 0.6)	0.9 (0.7, 1.1)
Implant	0.5 (0.5, 0.5)	0.7 (0.6, 0.8)	1.0 (1.0, 1.0)	1.3 (1.1, 1.4)	0.3 (0.2, 0.4)	0.5 (0.4, 0.7)
Short-acting method	3.1 (3.1, 3.2)	3.6 (3.4, 3.8)	5.4 (5.4, 5.5)	6.3 (6.0, 6.6)	1.9 (1.7, 2.1)	2.5 (2.1, 2.9)
Injectable	1.4 (1.4, 1.5)	1.7 (1.6, 1.8)	2.4 (2.4, 2.5)	2.9 (2.7, 3.1)	0.7 (0.5, 0.8)	0.9 (0.7, 1.2)
Oral / patch / ring	1.7 (1.7, 1.8)	1.9 (1.8, 2.0)	3.0 (3.0, 3.1)	3.4 (3.2, 3.6)	1.2 (1.0, 1.4)	1.6 (1.2, 1.9)

© 2025 Bellerose M et al. *JAMA Network Open*.

5. Use later transition rather than never transition as sDID control group

We tested the impact of using “later treated” or “not yet treated” observations as the control group in our sDID analysis, meaning people who had a later transition from Medicare to dual enrollment, instead of “never treated” observations who remained continuously enrolled in Medicare alone. The results of this analysis were roughly equivalent in direction and magnitude to the main analysis results (eTable 14).

eTable 14. sDID Results Using Later Transition as Control Group

	Percentage point change (95% CI)	
	12-month average	12 months after transition
Any method	2.7 (2.5, 2.9)	3.9 (3.5, 4.3)
Permanent method	0.1 (0.0, 0.1)	0.1 (0.1, 0.2)
Long-acting method	0.8 (0.7, 0.9)	1.4 (1.2, 1.6)
IUD	0.5 (0.4, 0.6)	0.8 (0.7, 1.0)
Implant	0.3 (0.3, 0.4)	0.6 (0.5, 0.7)
Short-acting method	1.9 (1.7, 2.2)	2.6 (2.3, 3.0)
Injectable	0.7 (0.6, 0.9)	1.0 (0.8, 1.3)
Oral / patch / ring	1.2 (1.1, 1.4)	1.6 (1.3, 1.9)

6. Run balanced sDID model

In the main analysis, used an unbalanced panel to maintain a large sample size for better power. However, in an sDID model, the compositions of the cohorts can change over time depending on the amount of time that an individual is “exposed”[11] – in this case the length of time that someone has Medicare prior to their transition. Therefore, in eTable 15, we test the impact of using a balanced panel restricted to people with 12 months of pre- and post-transition data. These results were slightly smaller in magnitude compared to the main analysis results and had wider confidence intervals due to the smaller sample size (n=42,412 women with 1,060,300 monthly observations) but remained significant.

eTable 15. sDID Results Using Balanced sDID Model

	Percentage point change (95% CI)	
	12-month average	12 months after transition
Any method	1.9 (1.4, 2.3)	3.1 (2.4, 3.9)
Permanent method	0.1 (0.0, 0.2)	--
Long-acting method	0.5 (0.3, 0.7)	1.0 (0.6, 1.4)
IUD	0.4 (0.2, 0.6)	0.8 (0.4, 1.1)
Implant	0.1 (0.0, 0.2)	--
Short-acting method	1.4 (0.9, 1.8)	2.2 (1.5, 2.8)
Injectable	0.6 (0.3, 0.9)	0.8 (0.4, 1.2)
Oral / patch / ring	0.8 (0.5, 1.2)	1.4 (0.9, 1.9)

© 2025 Bellerose M et al. *JAMA Network Open*.

7. Compare results to two-way fixed effects model

To test the robustness of our results, we tested a standard two-way fixed effects difference-in-differences design with individual and month fixed effects. The results were similar to the main analysis (eTable 16).

eTable 16. Two-Way Fixed-Effects Model

	Percentage point change (95% CI) 12-month average
Any method	3.5 (3.5, 3.6)
Permanent method	0.4 (0.4, 0.4)
Long-acting method	1.0 (1.0, 1.1)
IUD	0.6 (0.6, 0.6)
Implant	0.5 (0.5, 0.5)
Short-acting method	2.3 (2.2, 2.3)
Injectable	1.0 (1.0, 1.1)
Oral / patch / ring	1.3 (1.2, 1.3)

© 2025 Bellerose M et al. *JAMA Network Open*.

Contraceptive method switching

8. Switching between short-acting and long-acting or permanent methods

In eTable 17, we assess the impact of a Medicare to dual transition on contraceptive method switching. We did not include this analysis in the main results, as the sample of women actively using a contraceptive method before and after a transition was too small for adequate power. In the 12 months prior to a transition from Medicare alone to dual enrollment, 1.9% of women actively using any short-acting contraceptive method switched to a long-acting or permanent method per month. In the 12 months following a transition from Medicare alone to dual enrollment, this percentage increased to 3.0% per month. Gaining contraceptive coverage through dual enrollment led to a 1.0 percentage points (95% CI 0.3, 1.8) increase in switching from a short to a long-acting or permanent method. We did not find a significant effect of gaining coverage on transitioning from a long-acting method to a short-acting method.

eTable 17. Association of a Transition From Medicare to Dual Enrollment on Contraceptive Method Switching Among Women Using a Contraceptive Method

	Pre-transition, % (95% CI)		Post-transition, % (95% CI)		Percentage point change (95% CI)	
	12-month average	Month before transition	12-month average	12 months after transition	12-month average	12 months after transition
Switch from short-acting to long-acting or permanent method	1.9 (1.7, 2.0)	2.5 (2.1, 3.0)	2.8 (2.6, 2.9)	3.0 (2.3, 3.6)	0.6 (0.2, 1.1)	1.0 (0.3, 1.8)
Switch from long-acting method to short-acting method	1.2 (1.0, 1.3)	1.6 (1.2, 2.0)	1.6 (1.5, 1.7)	1.6 (1.1, 2.0)	0.1 (-0.3, 0.5)	0.3 (-0.3, 0.9)

© 2025 Bellerose M et al. *JAMA Network Open*.

Placebo Tests

9. Test alternative transitions (dual to Medicare alone, Medicaid alone to dual)

To increase our confidence that the observed effects resulted from gaining contraceptive coverage through a transition from Medicare alone to dual enrollment rather than from experiencing any transition in insurance coverage, we tested two alternative transitions: dual enrollment to Medicare alone and Medicaid alone to dual enrollment. We expected that a dual enrollment to Medicare alone transition would lead to a decline in contraceptive use as dual enrolled women have access to coverage of all contraceptive methods without cost sharing while Medicare enrolled women are subject to cost-sharing for short, long-acting, and permanent methods. We expected that a Medicaid alone to dual enrollment transition would not lead to a significant change in contraceptive use, as women enrolled in both insurance types have access to coverage of all contraceptive methods without cost-sharing. Our results supported the first hypothesis; experiencing a dual enrollment to Medicare transition led to a 1.4 percentage point decline (95% CI -1.9, -1.0) in any contraceptive use, with the largest decline in use of short-acting methods at of 1.1 percentage points (95% CI -1.5, -0.7) (eTable 18). Our results partially supported the second hypothesis; experiencing a Medicaid to dual enrollment transition did not have a significant effect on use of permanent methods, long-acting methods, or implants, but led to a significant increase in use of oral contraceptives, patches, and rings. This increase could be the result of greater interaction with the healthcare system upon new enrollment in Medicare, for example addressing contraceptive needs during an initial Medicare "welcome visit" with a primary care provider.

eTable 18. sDID Results Showing Association of Alternative Transitions With Contraceptive Use

	Pre-transition use, % (95% CI)		Post-transition use, % (95% CI)		Percentage point change (95% CI)	
	12-month average	Month before transition	12-month average	12 months after transition	12-month average	12 months after transition
Dual to Medicare						
Any method	12.8 (12.6, 12.9)	12.4 (12.0, 12.7)	13.3 (13.2, 13.5)	12.3 (11.7, 13.0)	-0.8 (-1.0, -0.5)	-1.4 (-1.9, -1.0)
Permanent method	3.8 (3.8, 3.9)	3.7 (3.5, 3.9)	3.9 (3.8, 4.0)	3.7 (3.3, 4.1)	-0.1 (-0.1, -0.1)	-0.2 (-0.3, -0.2)
Long-acting method	4.3 (4.2, 4.3)	4.4 (4.2, 4.6)	5.3 (5.2, 5.4)	5.6 (5.1, 6.0)	-0.2 (-0.4, -0.1)	-0.4 (-0.6, -0.1)
IUD	3.3 (3.3, 3.4)	3.3 (3.1, 3.5)	3.9 (3.8, 4.0)	4.1 (3.7, 4.5)	-0.1 (-0.2, 0.0)	-0.2 (-0.4, 0.0)
Implant	1.1 (1.0, 1.1)	1.2 (1.1, 1.3)	1.6 (1.5, 1.6)	1.5 (1.3, 1.8)	-0.2 (-0.2, -0.1)	-0.2 (-0.4, -0.1)
Short-acting method	5.2 (5.1, 5.2)	4.8 (4.5, 5.0)	4.5 (4.4, 4.6)	3.5 (3.1, 3.9)	-0.6 (-0.8, -0.3)	-1.1 (-1.5, -0.7)
Injectable	2.2 (2.2, 2.3)	2.1 (1.9, 2.2)	1.9 (1.8, 1.9)	1.2 (1.0, 1.5)	-0.2 (-0.4, -0.1)	-0.3 (-0.6, -0.1)
Oral / patch / ring	3.0 (2.9, 3.1)	2.7 (2.6, 2.9)	2.7 (2.6, 2.8)	2.3 (2.0, 2.6)	-0.3 (-0.5, -0.1)	-0.7 (-1.1, -0.4)

© 2025 Bellerose M et al. *JAMA Network Open*.

Medicaid to dual transition						
Any method	11.6 (11.4, 11.8)	12.6 (12.0, 13.2)	15.0 (14.8, 15.2)	16.4 (15.6, 17.1)	1.3 (0.8, 1.7)	1.9 (1.1, 2.6)
Permanent method	0.9 (0.8, 0.9)	1.1 (0.9, 1.3)	1.5 (1.4, 1.5)	1.8 (1.6, 2.1)	0.0 (-0.0, 0.1)	0.1 (-0.1, 0.2)
Long-acting method	4.2 (4.1, 4.4)	5.1 (4.7, 5.4)	6.2 (6.1, 6.3)	7.1 (6.6, 7.6)	0.2 (0.0, 0.4)	0.4 (0.0, 0.8)
IUD	2.5 (2.4, 2.6)	3.0 (2.7, 3.3)	3.7 (3.6, 3.8)	4.2 (3.8, 4.7)	0.1 (-0.1, 0.2)	0.2 (-0.1, 0.5)
Implant	1.9 (1.8, 2.0)	2.2 (2.0, 2.5)	2.7 (2.7, 2.8)	3.1 (2.8, 3.5)	0.2 (0.0, 0.3)	0.3 (-0.0, 0.5)
Short-acting method	6.9 (6.7, 7.0)	7.0 (6.5, 7.4)	7.9 (7.8, 8.0)	8.0 (7.4, 8.5)	1.0 (0.5, 1.4)	1.3 (0.6, 1.9)
Injectable	3.8 (3.7, 3.9)	3.6 (3.3, 4.0)	3.8 (3.7, 3.9)	3.8 (3.4, 4.2)	-0.0 (-0.3, 0.3)	0.1 (-0.4, 0.5)
Oral / patch / ring	3.1 (3.0, 3.2)	3.4 (3.1, 3.7)	4.1 (4.0, 4.2)	4.2 (3.8, 4.6)	1.0 (0.7, 1.3)	1.1 (0.6, 1.7)

eFigure 12. Event Study Plots of Contraceptive Use After Dual Enrollment to Medicare Transition (Left) and Medicaid to Dual Transition (Right), 2016 to 2020

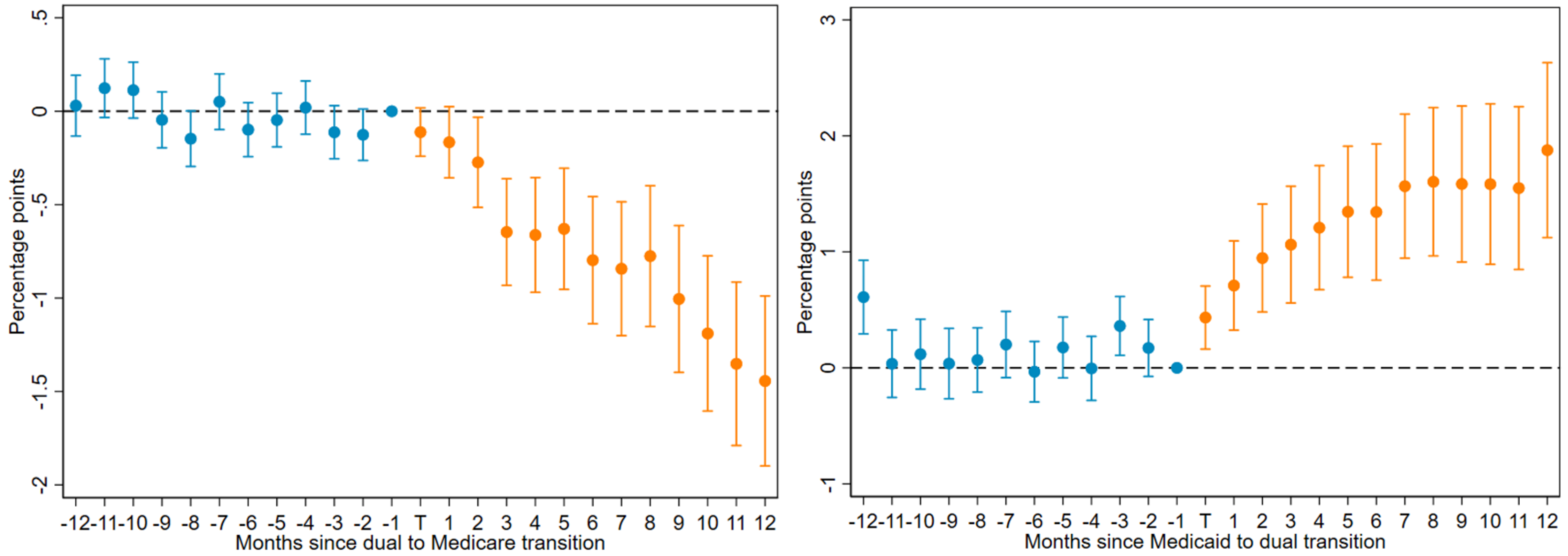

© 2025 Bellerose M et al. *JAMA Network Open*.

10. Pap smears

To further evaluate whether observed effects were the result of gaining contraceptive coverage rather than experiencing any transition in insurance type, we performed a placebo test looking at the impact of a Medicare to dual enrollment on receipt of Papanicolaou tests ("pap smears"). A pap smear is a procedure recommended every three years for women ages 21 through 49 where cells from the cervix are tested to detect cervical cancer. Unlike contraceptives, pap smears are fully covered by TM, MA, and Medicaid without cost sharing. Therefore, we would not expect to see an impact of a transition on receipt of a pap smear. Codes used to identify pap smears are provided in eTable 19. In line with that hypothesis, we did not find a significant effect (eTable 20).

eTable 19. Pap Smear Diagnostic Codes

ICD-10	CPT	HCPCS
Z01411	88141-88145	P3000
Z01419	88147-88148	P3001
Z0142	88150-88158	G0101
Z124	88164-88167	G0123
Z1272	88174-88175	G0124
R87614		G0141
		G0143
		G0144
		G0145
		G0147
		G0148
		Q0091

eTable 20. sDID Results Showing Association of Transition From Medicare to Dual Enrollment With Pap Smear Receipt

	Pre-transition use, % (95% CI)		Post-transition use, % (95% CI)		Percentage point change (95% CI)	
	12-month average	Month before transition	12-month average	12 months after transition	12-month average	12 months after transition
Pap smear	0.1 (0.1, 0.1)	0.3 (0.2, 0.3)	0.2 (0.1, 0.3)	0.3 (0.2, 0.4)	0.1 (-0.1, 0.2)	0.1 (-0.1, 0.2)

eReferences.

1. Social Security Administration. *Annual Statistical Report on the Social Security Disability Insurance Program, 2022. Table 6.* 2023. https://www.ssa.gov/policy/docs/statcomps/di_asr/2022/index.html

2. Kaiser Family Foundation. Medicaid Income Eligibility Limits for Adults as a Percent of the Federal Poverty Level. Updated January 1, 2021. Accessed April 4, 2022, https://www.kff.org/health-reform/state-indicator/medicaid-income-eligibility-limits-for-adults-as-a-percent-of-the-federal-poverty-level

3. Musumeci M, Orgera K. Supplemental security income for people with disabilities: Implications for Medicaid. *KFF Issue Brief San Francisco, CA: Kaiser Family Foundation*. 2021;

4. Centers for Medicare & Medicaid Services (CMS). *Medicare Savings Programs*. 2025. Accessed April 4, 2025. https://www.medicare.gov/basics/costs/help/medicare-savings-programs

5. DQAtlas. Beneficiary Information: Eligibility Group Code. https://www.medicaid.gov/dq-atlas/welcome

6. Cole SR, Hernán MA. Constructing inverse probability weights for marginal structural models. *American journal of epidemiology*. 2008;168(6):656-664.

7. Desai RJ, Franklin JM. Alternative approaches for confounding adjustment in observational studies using weighting based on the propensity score: a primer for practitioners. *bmj*. 2019;367

8. Nead KT, Hinkston CL, Wehner MR. Cautions when using race and ethnicity in administrative claims data sets. American Medical Association; 2022:e221812-e221812.

9. Healthline. Does Medicare Cover Birth Control? January 14, 2021. https://www.healthline.com/health/medicare/does-medicare-cover-birth-control

10. Elze MC, Gregson J, Baber U, et al. Comparison of propensity score methods and covariate adjustment: evaluation in 4 cardiovascular studies. *Journal of the American College of Cardiology*. 2017;69(3):345-357.

11. Callaway B, Sant'Anna PHC. Difference-in-Differences with multiple time periods. *Journal of Econometrics*. 2021/12/01/ 2021;225(2):200-230. doi:https://doi.org/10.1016/j.jeconom.2020.12.001

© 2025 Bellerose M et al. *JAMA Network Open*.
