# Supplementary material for: Coverage Gaps and Contraceptive Use Among Medicare Enrollees With Disabilities
Source: JAMA Netw Open. 2025 Jun 25;8(6):e2517718. doi: 10.1001/jamanetworkopen.2025.17718 (PMC12199052; doi:10.1001/jamanetworkopen.2025.17718)
Supplement: Supplement 2. — Data Sharing Statement [file jamanetwopen-e2517718-s002.pdf]

# Data Sharing Statement

Bellerose. Coverage Gaps and Contraceptive Use Among Medicare Enrollees With Disabilities. *JAMA Netw Open*. Published June 25, 2025. doi:10.1001/jamanetworkopen.2025.17718

**Data**
**Data available:** No

**Additional Information**
**Explanation for why data not available:** The data used in this study were provided by the Centers for Medicare and Medicaid Services (CMS) under Data Use Agreement and contain protected health information.
